# Changes in the expression of cancer- and metastasis-related genes and proteins after metformin treatment under different metabolic conditions in endometrial cancer cells

**DOI:** 10.1016/j.heliyon.2023.e16678

**Published:** 2023-05-25

**Authors:** Carsten Lange, Jana Brüggemann, Theresa Thüner, Julia Jauckus, Thomas Strowitzki, Ariane Germeyer

**Affiliations:** Department of Gynecological Endocrinology and Fertility Disorders, University Women's Hospital, Ruprecht-Karls University of Heidelberg, Im Neuenheimer Feld 440, 69120 Heidelberg, Germany

**Keywords:** Endometrial cancer, Metformin, Hyperinsulinemia, Hyperglycemia

## Abstract

**Research question:**

Hyperinsulinemia and elevated estrogen levels are known risk factors for endometrial cancer (EC) development and are associated with obesity, type 2 diabetes mellitus (T2DM), insulin resistance, among others. Metformin, an insulin-sensitizing drug, displays anti-tumor effects in cancer patients, including EC, but the mechanism of action is still not completely understood. In the present study, the effects of metformin on gene and protein expression were investigated in pre- and postmenopausal EC *in vitro* models in order to identify candidates that are potentially involved in the drug's anti-cancer mechanism.

**Design:**

After treating the cells with metformin (0.1 and 1.0 mmol/L), changes in the expression of >160 cancer- and metastasis-related gene transcripts were evaluated with RNA arrays. A total of 19 genes and 7 proteins were selected for a follow-up expression analysis, including further treatment conditions, in order to evaluate the influence of hyperinsulinemia and hyperglycemia on metformin-induced effects.

**Results:**

Changes in the expression of BCL2L11, CDH1, CDKN1A, COL1A1, PTEN, MMP9 and TIMP2 were analyzed on gene and protein level. The consequences resulting from the detected expression changes as well as the influence of varying environmental influences are discussed in detail. With the presented data, we contribute to a better understanding of the direct anti-cancer activity of metformin as well as its underlying mechanism of action in EC cells.

**Conclusions:**

Although further research will be necessary to confirm the data, the influence of different environmental settings on metformin-induced effects could be highlighted with the presented data. Additionally, gene and protein regulation were not similar in the pre- and postmenopausal *in vitro* models.

## Graphical abstract

**Figure:**
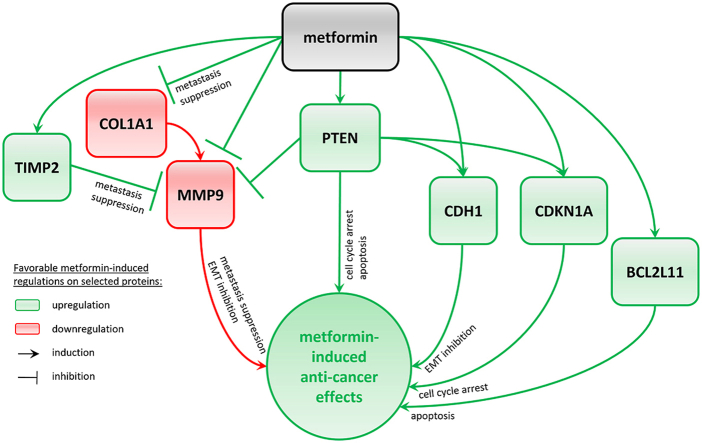

## 1 Introduction

Obesity, hyperglycemia and related type 2 diabetes mellitus (T2DM), as well as other associated diseases such as polycystic ovary syndrome (PCOS) and insulin resistance lead to hyperinsulinemia and/or elevated estrogen levels, which are known risk factors for the development and progression of endometrial cancer (EC) [[1], [2], [3], [4]].

Metformin, an insulin-sensitizing biguanide agent used in T2DM therapy [5], displayed anti-cancer effects in numerous *in vitro* studies, but also in diabetic patients with EC and other cancer entities [[6], [7], [8], [9], [10], [11], [12]]. During T2DM treatment, metformin reduces circulating glucose and insulin levels via a blockage of gluconeogenesis in the liver and via improvement of peripheral insulin sensitivity by opposing the action of glucagon, increasing glucose uptake in the muscle while decreasing glucose absorption in the small intestine, and inhibiting lipolysis in adipose tissue [[13], [14], [15], [16]]. Metformin's anti-cancer effects target signaling pathways related to cellular growth and proliferation via activation of 5′ adenosine monophosphate-activated protein kinase (AMPK), leading to a subsequent induction of tumor suppressors as well as a regulation of growth-related pathways [17]. Additionally, metformin acts independent of AMPK, e.g. via ras-related guanosine triphosphate hydrolases (Rag GTPases) or mitochondrial respiratory-chain complex 1 [[17], [18], [19], [20]]. However, the mechanism of metformin's direct anti-tumor activity is still not completely understood.

In the present study, the effects of metformin on gene and protein expression were investigated in the human EC cell lines HEC-1A and Ishikawa in order to identify candidates that are involved in the drug's anti-cancer mechanism in EC. HEC-1A and Ishikawa cells represent different EC *in vitro* model systems, namely a pre- (Ishikawa) and a postmenopausal (HEC-1A) model. HEC-1A cells are characterized by a poor expression of estrogen receptor α (ERα) [21,22] and maintain low cellular estrogen sensitivity due to the expression of ERβ and G protein-coupled estrogen receptor 1 (GPER) [23]. Ishikawa cells are defined by an intact expression of ERα/β, GPER and progesterone receptor (PR), equipping the cell line with an unaffected sensitivity to β-estradiol (E2) [12,[23], [24], [25], [26]]. The design of the current *in vitro* study took different clinical settings into account that are associated with EC development and progression. Firstly, insulin supplementation was incorporated into the study in order to implement a model for insulin resistance and related hyperinsulinemia, both often observed in obese, prediabetic or PCOS patients that are prone to EC development [27,28]. Secondly, cells were treated in the presence of normal (5.5 mmol/L) or elevated glucose concentrations (17.0 mmol/L) to be able to evaluate the influence of hyperglycemia on metformin-induced effects. Thirdly, cells were treated in the presence of E2 in order to mimic increased estrogen levels that contribute to EC development and progression and, like hyperinsulinemia, result from aforementioned diseases [1,2,29].

After treating the cells with metformin for 7 d, changes in the expression of >160 cancer- and metastasis-related gene transcripts were evaluated with the help of RNA arrays, of which 19 candidates, namely *APC*, *BCL2L11*, *CASP8*, *CDH1*, *CDKN1A*, *CEACAM1*, *COL1A1*, *CTNNA1*, *IGF1*, *PTEN*, *RAC1*, *TGFB1*, *MMP2/9/10* and *TIMP1/2/3/4*, were selected for a follow-up quantitative real-time PCR (qPCR) analysis, including further treatment conditions. Afterwards, changes on protein level were additionally investigated by Western blot analysis for those selected 7 candidates that had shown the most prominent and favorable changes in gene expression analysis, namely BCL2L11, CDH1, CDKN1A, COL1A1, PTEN, MMP9 and TIMP2. The consequences for the cells resulting from the detected gene and protein expression changes as well as the influence of varying environmental influences are discussed in detail.

With the presented data, several genes and proteins were identified that might be involved in the direct anti-cancer mechanism of metformin in EC. However, further pathway analysis will be necessary in order to identify upstream regulators and downstream targets of the identified candidate genes and proteins, e.g. with regards to promising candidates such as the apoptosis-related BCL2L11 protein, the cell cycle mediator CDKN1A, or metastasis-associated MMP9 and TIMP2.

## 2 Materials and methods

### 2.1 Cell culture and metformin treatment

The human EC cell lines HEC-1A (estrogen-nonresponsive adenocarcinoma (postmenopausal model); HTB112, ATCC, Manassas, VA, USA) [21,22] and Ishikawa (estrogen-responsive adenocarcinoma (premenopausal model); 99040201, Sigma-Aldrich, Munich, Germany) [24,25] were cultured in phenol red-free Eagle's minimal essential medium (MEM, 5.5 mmol/L glucose, equivalent to 100 mg/dL; Sigma-Aldrich) supplemented with 10% (v/v) charcoal-stripped fetal bovine serum (hormone-reduced FBS; Gibco, Waltham, MA, USA), 1% (v/v) stable glutamine (Sigma-Aldrich), and 100 μg/mL streptomycin/100 U/mL penicillin G (Gibco) at 37 °C and 5% CO_2_ in a humidified atmosphere. Cell lines were routinely checked for mycoplasma with the MycoAlert mycoplasma detection kit (Lonza, Basel, Switzerland) according to the manufacturer's protocol. Cells were passaged with 0.25% (v/v) trypsin/ethylenediaminetetraacetic acid (EDTA; Gibco) once a week and the medium was changed every 2–3 d. Experiments were carried out in 6-well plates with a seeding density of 2.0 × 10^4^ cells in 2 mL medium in duplicates. After seeding, cells were allowed to attach and grow for 24 h before treatment.

Cells were treated with either 0.1 or 1.0 mmol/L metformin (100 mmol/L stock in H_2_O; Sigma-Aldrich), 100 ng/mL insulin (10.0 μg/mL stock in phosphate-buffered saline (PBS); Sigma-Aldrich) or a combination of metformin and insulin under normo- (5.5 mmol/L glucose) or hyperglycemic conditions (17.0 mmol/L glucose, equivalent to 300 mg/dL) in the presence of 10 nmol/L E2 (all purchased from Sigma-Aldrich) in phenol-red-free medium for 7 d with medium changes and renewed treatments every 2–3 d. The selected metformin concentrations induced no loss of cellular viability in the ATP, and a mild to moderate loss of cellular viability in the MTT (3-(4,5-dimethylthiazol-2-yl)-2,5-diphenyltetrazolium bromide) viability assays under similar experimental conditions in an *in vitro* study recently published by our group [12] and therefore ensured to maintain subtoxic conditions throughout the duration of the treatment. An insulin concentration of ≥50 ng/mL is well established in *vitro* studies in the field of endocrinology and diabetes research in order to mimic hyperinsulinemia [[30], [31], [32], [33]] and has also been used by our group before [[34], [35], [36]]. Control cells were treated with substance-free medium supplemented with 10 nmol/L E2 as well as H_2_O and PBS (Sigma-Aldrich) as vehicles.

### 2.2 RNA extraction and cDNA synthesis

After 7 d, the culture medium was removed and the cells were washed with PBS twice. RNA was extracted from the aqueous phase after addition of 500 μL/well TRIzol reagent (Invitrogen, Waltham, MA, USA) and 200 μL chloroform (Sigma-Aldrich). Afterwards, total RNA was precipitated with 500 μL isopropanol containing 1,6 μL GlycoBlue co-precipitant (Invitrogen) at −20 °C for 2 h, washed with 1 mL 75% (v/v) ethanol, and resuspended in 100 μL nuclease-free H_2_O (Sigma-Aldrich). RNA concentration was detected with a NanoDrop spectrophotometer (ND-1000; Thermo Fisher) and samples were stored at −80 °C until cDNA synthesis.

Reverse transcription of 1.0 μg of the collected RNA was carried out with the reverse transcription system kit (Promega, Fitchburg, WI, USA) using avian myeloblastosis virus (AMV) reverse transcriptase according to the manufacturer's protocol in order to synthesize complementary DNA (cDNA). Generated cDNA samples were stored at −20 °C until gene expression analysis.

### 2.3 Gene expression analysis with RNA arrays and real-time PCR

For the identification of target genes, differential gene expression of >160 genes related to cancer and metastasis was investigated with the TaqMan “Human Molecular Mechanisms of Cancer” (4418806) and “Human Tumor Metastasis” (4418743; both from Thermo Fisher, Waltham, MA, USA) RNA arrays in a first step. Two samples were selected for the screening in each cell line: 1.0 mmol/L metformin compared to untreated control cells under hyperglycemic conditions.

The expression of identified target genes was evaluated in a follow-up qPCR analysis including additional treatment conditions (see section 4.1). For qPCR analysis, cDNA was diluted in nuclease-free water (Promega) and mixed with the designated TaqMan gene expression assay diluted in TaqMan universal PCR master mix (no uracil *N*-glycosylase (UNG); Applied Biosystems, Foster City, CA, USA) to a final volume of 5 μL. The mixture was then pipetted into 96-well PCR plates (Applied Biosystems), centrifuged and data were recorded with a qPCR system (7500 Fast; Applied Biosystems) using the following protocol: 95 °C for 10 min as initial step followed by 40 cycles with 95 °C for 15 s and 60 °C for 1 min. TaqMan gene expression assays for the following genes were used in the experiments (all from Applied Biosystems): *APC* (adenomatous polyposis coli; Hs00181051_m1), *BCL2L11* (Bcl-2-like protein 11, BIM; Hs00197982_m1), *CASP8* (caspase 8; Hs01018151_m1), *CDH1* (cadherin 1, *E*-cadherin; Hs01023894_m1), *CDKN1A* (cyclin-dependent kinase inhibitor 1 A, p21^Waf1/Cip1^; Hs00355782_m1), *CEACAM1* (carcinoembryonic antigen-related cell adhesion molecule 1, BGP1, CD66a; Hs00989783_m1), *COL1A1* (collagen type 1 alpha 1; Hs00164004_m1), *CTNNA1* (catenin alpha-1; Hs00944792_mH), *IGF1* (insulin-like growth factor 1; Hs01547656_m1), *MMP2/9/10* (matrix metalloproteinase 2/9/10, gelatinase A/B/stromelysin 2; Hs01548727_m1, Hs00234579_m1, Hs00233987_m1), *PTEN* (phosphatase and tensin homolog; Hs01920652_s1), *RAC1* (ras-related C3 botulinum toxin substrate 1; Hs01025984_m1), *TGFB1* (transforming growth factor beta 1; Hs00998133_m1), and *TIMP1/2/3/4* (tissue inhibitor of metalloproteinases 1/2/3/4; Hs00171558_m1, Hs00234278_m1, Hs00165949_m1, Hs00162784_m1). Threshold cycle (C_T_) values were determined with the 7500 software (Applied Biosystems) and used for relative expression analysis (ΔC_T_ method) against 18 S ribosomal RNA (hs99999901_s1; Applied Biosystems) as the reference. Data are presented as expression levels relative to the expression in untreated reference cells under normo- and hyperglycemic conditions for visualization of the metformin/insulin effects and as expression levels of hyperglycemic samples relative to the expression of equally treated samples under normoglycemia to visualize the glucose effect using the 2^–ΔΔCT^ method (fold-change, FC).

### 2.4 Protein extraction and BCA assay

After 7 d, the culture medium was removed and the cells were washed with PBS twice. Then, cells were lysed with 200 μL/well ice-cold RIPA buffer (Thermo Fisher, Waltham, MA, USA) supplemented with protease inhibitor cocktail (PIC; Roche, Basel, Switzerland). After gentle shaking on ice for 5 min, samples were collected and centrifuged at 14,000 g at 4 °C for 15 min. Protein extracts were stored at −80 °C until Western blot analysis.

Protein concentrations were determined with the bicinchoninic acid (BCA) assay (Thermo Fisher) according to the manufacturer's protocol with the help of a microplate reader (AR2001; Anthos Microsystems, Friesoythe, Germany) at λ = 570 nm.

### 2.5 Protein analysis via western blotting

For Western blot analysis, equal amounts of total protein (10–35 μg) were heated to 85 °C for 3 min in Laemmli buffer [37] (0.5 mol/L dithiothreitol (DTT) instead of β-mercaptoethanol), separated by SDS-PAGE using pre-cast Tris-glycine gradient gels (8–16%; Invitrogen) and transferred to polyvinylidene difluoride (PVDF) membranes (Bio-Rad, Karlsruhe, Germany) with a transfer system (*Trans*-blot Turbo; Bio-Rad). Transferred total proteins were detected with the no-stain protein labeling reagent (Invitrogen) according to the manufacturer's protocol and were used as the loading control. Afterwards, membranes were blocked with 5% (w/v) non-fat milk (Merck, Darmstadt, Germany) in Tris buffer containing 0.05% (v/v) Tween-20 (TBST; Carl Roth, Karlsruhe, Germany) at room temperature for 2 h. After washing for three times with TBST for 10 min, the membranes were incubated with primary rabbit antibodies at 4 °C overnight in TBST supplemented with 5% (w/v) bovine serum albumin (BSA; Biomol, Hamburg, Germany) directed against the following human proteins: BCL2L11 (2933), CDH1 (3195), MMP9 (3852) PTEN (9559), TIMP2 (5738; all from Cell Signaling, Leiden, Netherlands), CDKN1A (ab109520) and COL1A1 (ab138492; both from Abcam, Cambridge, UK) (Fig. S3). Afterwards, membranes were washed again and incubated with goat anti-rabbit IgG secondary antibody conjugated with horseradish peroxidase (HRP; 7074; Cell Signaling) at room temperature for 4 h. Chemiluminescence was detected after washing and subsequent incubation with enhanced chemiluminescence (ECL) substrate solution (Cytiva, Marlborough, MA, USA) for 2 min in the dark and was visualized with an imaging system (iBright FL1500; Thermo Fisher). Semi-quantitative, densitometric Western blot analysis has been done with the ImageJ software [38,39]. Data are presented as expression levels normalized with total protein expression and relative to the expression in untreated reference cells under normoglycemic conditions (fold-change).

### 2.6 Statistical analysis

RNA arrays were only performed once in order to screen for genes involved in cancer mechanisms and tumor metastasis that were affected by metformin treatment, and thus no statistical analysis was performed with the resulting data.

For qPCR analysis, ΔC_T_ and ΔΔC_T_ values were calculated according to equations (1), (2), respectively:

(1) ΔC_T_ = C_T, GOI_ – C_T, 18S_

and

(2) ΔΔC_T_ = ΔC_T, treatment_ – ΔC_T, NG/HG control_

A mixed effects model analysis was performed with the ΔC_T_ values followed by Tukey's (comparing metformin/insulin effects under identical glucose conditions) or Šídák's (comparing identical treatments between different glucose levels) multiple comparison *post-hoc* tests. In order to ensure reliability of the qPCR data, samples with C_T_ values ≥ 34.0 were excluded due to their low expression levels.

For western blotting, mixed effects model analysis and subsequent Tukey's or Šídák's multiple comparison *post-hoc* test were performed with protein expression levels normalized to the total protein amount and relative to protein levels in untreated cells under normoglycemic conditions.

Real-time PCR and Western blot data were presented as aligned dot plots including the means of at least three independent experiments. Statistical analyses were carried out with the help of Prism 9 (GraphPad Software, La Jolla, CA, USA). A value of *p* ≤ 0.05 was considered statistically significant; *p* < 0.1 were additionally mentioned in the text and *p* < 0.15 were displayed as values in the plots. Unless stated otherwise, *p* values mentioned in the text refer to the untreated normo- or hyperglycemic control sample. Single missing samples and values appeared due to random reasons (e.g. cell handling errors, low RNA quality, and pipetting or other errors during cDNA synthesis, qPCR, BCA assay, SDS PAGE or western blotting).

## 3 Results

### 3.1 Screening for metformin-regulated target genes in transcriptome analysis

**Fig. 1:**
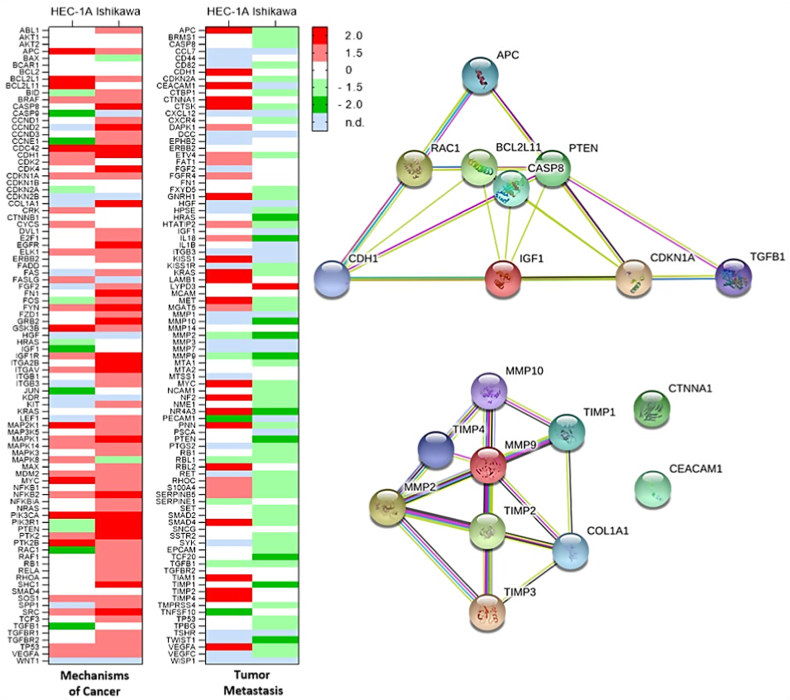
Changes in the expression of >160 genes after treatment of HEC-1A and Ishikawa cells with metformin in a hyperglycemic environment for 7 d. Cells were treated with 1.0 mmol/L metformin under hyperglycemic conditions (17.0 mmol/L glucose) for 7 d and TaqMan RNA arrays were performed afterwards; n = 1. (**a**) Heat map of the changes in gene expression in the TaqMan “Human Molecular Mechanisms of Cancer” and “Human Tumor Metastasis” RNA arrays for HEC-1A and Ishikawa cells. (**b**) Networks of 19 genes that were selected for a more detailed follow-up qPCR analysis including further treatment conditions.

From >160 different genes investigated in the TaqMan “Human Molecular Mechanisms of Cancer” and “Human Tumor Metastasis” RNA arrays, 11 and 21 genes, respectively, showed a ≥ 2-fold up- or downregulation and were regulated by metformin in a favorable, tumor-suppressing way (Fig. 1 and Table 1). Further genes were ≥ 2-fold up- or downregulated due to metformin treatment, however, regulation was rather unfavorable and tumor-promoting (Table 1); these genes were not considered for further analyses.

**Table 1:** Metformin-induced changes in gene expression as detected after metformin treatment in transcriptomic analysis. HEC-1A and Ishikawa cells were treated with 1.0 mmol/L metformin under hyperglycemic conditions and gene expression was compared to untreated control cells. For the identification of target genes, the expression of >160 genes was investigated with the TaqMan “Human Molecular Mechanisms of Cancer” and “Human Tumor Metastasis” RNA arrays. Interesting target genes were ≥ 2-fold up- or downregulated in a favorable, tumor-suppressing way due to the metformin treatment.

Human Molecular Mechanisms of Cancer		
Fold-change	HEC-1A	Ishikawa
≤0.5	favorable:*CCNE1*, *IGF1*, *JUN*, *RAC1*, *TGFB1*	favorable: none
	unfavorable:*CASP9*	unfavorable: none
≥2.0	favorable:*APC*, *BCL2L11*	favorable:*CASP8*, *CDH1*, *ITGA2B*, *PTEN*
	unfavorable:*BCL2L1*, *CDC42*, *GSK3B*, *MAP2K1*, *MYC*, *PIK3CA*, *PTK2B*	unfavorable:*CCND2*, *CDC42*, *CDK4*, *COL1A1, EGFR*, *FN1, FYN*, *GRB2*, *IGF1R, ITGAV*, *MAPK1*, *NFKB2*, *PIK3CA*, *PIK3R1*, *PTK2*, *SHC1*, *SRC*
Human Tumor Metastasis		
**Fold-change**	**HEC-1A**	**Ishikawa**
≤0.5	favorable:*PECAM1*	favorable:*HRAS*, *IL18*, *MMP2*, *MMP9*, *MMP10*, *NR4A3*, *TCF20*, *TIMP1*, *TWIST1*
	unfavorable:*TNFSF10*	unfavorable:*PTEN*
≥2.0	favorable:*APC*, *CDH1*, *CEACAM1*, *CTNNA1*, *KISS1*, *NF2*, *PNN*, *RBL2*, *SMAD4*, *TIMP2*, *TIMP4*	favorable: none
	unfavorable*: CTSK*, *GNRH1*, *KRAS*, *LAMB1*, *MET*, *MYC*, *NR4A3*, *TIAM1*, *VEGFA*	unfavorable:*LYPD3*

From the aforementioned eligible 32 genes, a total of 19 different genes were selected for a follow-up qPCR analysis (mechanisms of cancer: *APC*, *BCL2L11*, *CASP8*, *CDH1*, *CDKN1A*, *COL1A1*, *IGF1*, *PTEN*, *RAC1*, *TGFB1*; tumor metastasis: (*APC*, *CDH1*), *CEACAM1*, *CTNNA1*, *MMP2/9/10*, *TIMP1/2/3/4*), including further treatment conditions.

Although *COL1A1* expression was not detected in HEC-1A cells and was upregulated in Ishikawa cells, which is rather unfavorable, the gene has been selected for the follow-up qPCR analysis, because it is directly connected to selected *MMP2/9/10* as well as indirectly related to selected *TIMP1/2/4*. More importantly, our group observed a favorable downregulation of COL1A1 expression in an analysis of the HEC-1A proteome after metformin treatment in a recent study [34]. Additionally, *TIMP3* has also been selected for further analysis due to the connection to the selected *MMP2/9/10* and *TIMP1/2/4* genes. Furthermore, *CDKN1A* has been selected for further analysis, because it was regulated in a favorable way in both cell lines, although upregulation was only 1.8-fold. An overview of selected genes/proteins and their relation to cancer and metastasis is given in Table 2.

**Table 2:** Genes selected for follow-up qPCR analysis and their function and relation to cancer as well as metastasis.

Gene	Protein	Function and Cellular Processes	Relation to Cancer and Metastasis
*APC*	APC	neg. reg. cell proliferation	tumor suppressor, mutation related to cancer
*BCL2L11*	BCL2L11, BIM^a^	pro-apoptotic (intrinsic)	downregulated in tumors
*CASP8*	CASP8	pro-apoptotic (extrinsic)	mostly suppresses tumor metastasis
*CDH1*	CDH1, *E*-cadherin^a^	pos. reg. cell adhesion (cell-cell)	mutation related to cancer, suppresses EMT and invasion
*CDKN1A*	CDKN1A, p21^Waf1/Cip1^^a^	inhibits G_1_/S cell cycle progression, induces DNA repair and G_2_ arrest	mostly suppresses tumor growth and differentiation
*CEACAM1*	CEACAM1, BGP1, CD66a	pos. reg. cell adhesion (cell-cell)	suppresses metastasis, tumor invasion and angiogenesis
*COL1A1*	COL1A1^a^	ECM component	promotes metastasis and invasion
*CTNNA1*	CTNNA1	pos. reg. cell adhesion (cell-cell)	downregulation associated with a variety of tumors
*IGF1*	IGF1	growth factor	related to cancer progression
*MMP2*	MMP2, gelatinase A	angiogenesis, ECM degradation	promotes EMT, invasion and metastasis
*MMP9*	MMP9, gelatinase B^a^	angiogenesis, ECM degradation	promotes EMT, invasion and metastasis
*MMP10*	MMP10, stromelysin 2	ECM degradation	promotes EMT, invasion and metastasis
*PTEN*	PTEN^a^	induction of cell cycle arrest, apoptosis and cellular senescence	tumor suppressor, mutation related to cancer
*RAC1*	RAC1	pos. reg. glucose uptake, cell migration and growth	promotes motility and metastasis
*TGFB1*	TGF-β1	growth factor	promotes tumor growth
*TIMP1*	TIMP1	ECM composition, wound healing, pos. Reg. cell proliferation, anti-apoptotic	ambiguous, but associated with poor prognosis in EC
*TIMP2*	TIMP2^a^	ECM composition, neg. reg. proliferation	suppresses metastasis
*TIMP3*	TIMP3	ECM composition, neg. reg. proliferation and angiogenesis, pro-apoptotic	suppresses tumor growth as well as metastasis and induces apoptosis
*TIMP4*	TIMP4	platelet aggregation, neg. reg. angiogenesis	suppresses invasion and metastasis

a Additional Western blot analysis on protein level.

Changes in the Expression of Selected Genes after Metformin Treatment under Different Metabolic Conditions in Real-Time PCR Analysis.

**Fig. 2:**
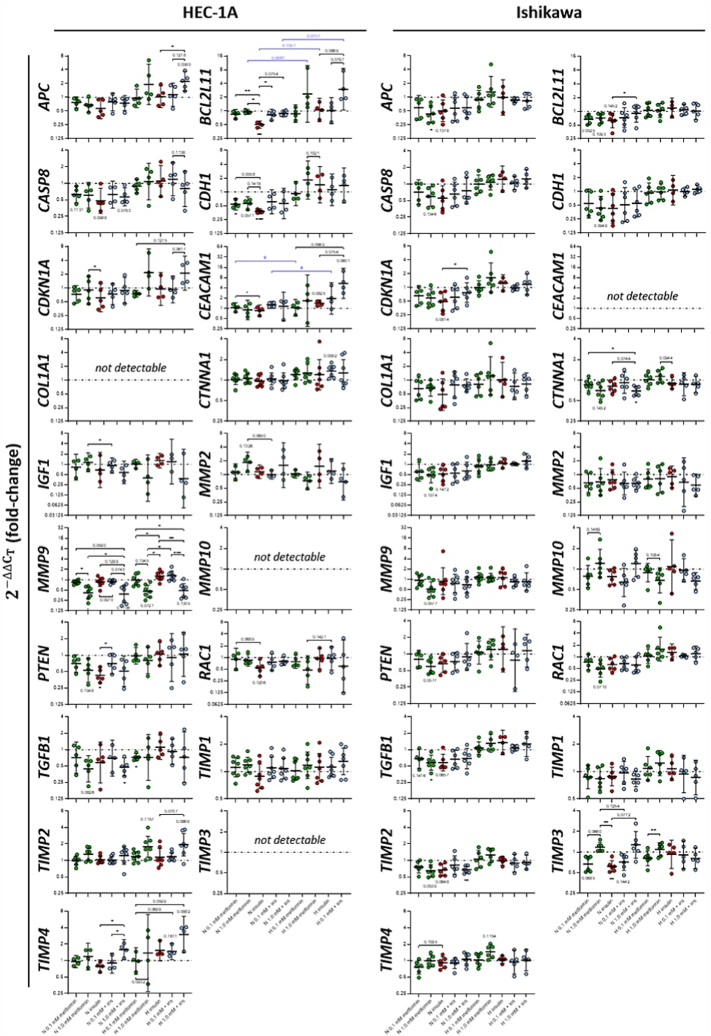
Changes in the expression of selected genes after treatment of HEC-1A and Ishikawa cells with metformin, insulin, or a combination of both substances for 7 d. Cells were treated with 0.1 and 1.0 mmol/L metformin (green), 100 ng/mL insulin (red) or a combination of metformin and insulin (blue) under normo- (5.5 mmol/L glucose) or hyperglycemic conditions (17.0 mmol/L glucose) for 7 d and real-time PCR analysis was performed afterwards. Expression levels were calculated relative to the expression in untreated reference cells under normo- and hyperglycemic conditions (fold-changes were set to 1.0 for these samples as indicated by a dotted line) using the 2^–ΔΔCT^ method (fold-change). Data presented as dot plots with geometric means of at least three independent experiments; n = 4–7. A mixed effects model analysis was performed with the ΔC_T_ values followed by Tukey's (analysis of metformin and insulin effects under identical glucose conditions) or Šídák's (analysis of glucose effects between identical treatments; see Figure S1) multiple comparison *post-hoc* tests; *p ≤ 0.05, **p ≤ 0.01 (metformin effect, black); ^#^*p* ≤ 0.05 (glucose effect, blue); *p* < 0.15 additionally displayed as values.

After selecting 19 target genes from the transcriptome analysis, a gene expression analysis has been carried out including further treatment conditions: metformin (0.1 and 1.0 mmol/L), insulin (100 ng/mL) and a combined administration, all under normo- (NG; 5.5 mmol/L) and hyperglycemic (HG; 17.0 mmol/L) conditions (Fig. 2). In the postmenopausal model cell line HEC-1A, *COL1A1*, *MMP10* and *TIMP3* genes could not be detected, whereas *CEACAM1* could not be found in the premenopausal Ishikawa cell line. Unless stated otherwise, *p* values refer to the untreated normo- or hyperglycemic control sample; *p* values < 0.1 were mentioned in the text.

In HEC-1A cells, metformin slightly downregulated gene expression of *APC* (FC_NG, 0.1_ = 0.78; FC_NG, 1.0_ = 0.68) and more substantially of *CASP8* (FC_NG, 0.1_ = 0.63; FC_NG, 1.0_ = 0.61) as well as *CDH1* (FC_NG, 0.1_ = 0.54, *p* = 0.029; FC_NG, 1.0_ = 0.56, *p* = 0.057) independent of the applied concentration in a normoglycemic environment. *MMP9* (FC_NG, 0.1_ = 0.85; FC_NG, 1.0_ = 0.42, *p* = 0.036 and *p* = 0.018 vs FC_NG, 0.1_), *PTEN* (FC_NG, 0.1_ = 0.70; FC_NG, 1.0_ = 0.53) and *TGFB1* (FC_NG, 0.1_ = 0.71; FC_NG, 1.0_ = 0.44, *p* = 0.083) expressions were lowered by metformin in a concentration-dependent manner. *MMP2* (FC_NG, 0.1_ = 1.12; FC_NG, 1.0_ = 1.86) was slightly upregulated after metformin administration only at 1.0 mmol/L, which was to a lesser extent also observed for *TIMP2* (FC_NG, 0.1_ = 1.00; FC_NG, 1.0_ = 1.29) expression. Only marginal effects of a metformin supplementation were observed for the expression of *BCL2L11*, *CDKN1A*, *CEACAM1*, *CTNNA1*, *IGF1*, *RAC1*, *TIMP1* and *TIMP4* (FC_NG, 0.1_ = 0.72–1.11; FC_NG, 1.0_ = 0.85–1.19).

Under hyperglycemic conditions, metformin downregulated *IGF1* (FC_HG, 0.1_ = 1.38; FC_HG, 1.0_ = 0.39), *MMP2* (FC_HG, 0.1_ = 1.04; FC_HG, 1.0_ = 0.73) and *MMP9* (FC_HG, 0.1_ = 0.98; FC_HG, 1.0_ = 0.46, *p* = 0.072) when applied at 1.0 mmol/L, while *RAC1* (FC_HG, 0.1_ = 0.78; FC_HG, 1.0_ = 0.48) as well as *TGFB1* (FC_HG, 0.1_ = 0.73, *p* = 0.028; FC_HG, 1.0_ = 0.72) expressions were additionally lowered at 0.1 mmol/L metformin. A substantial upregulation was detected with 1.0 mmol/L metformin for the following genes: *APC* (FC_HG, 0.1_ = 0.95; FC_HG, 1.0_ = 1.91), *BCL2L11* (FC_HG, 0.1_ = 0.88; FC_HG, 1.0_ = 2.38, *p* = 0.096 vs FC_NG, 1.0_), *CDH1* (FC_HG, 0.1_ = 0.93; FC_HG, 1.0_ = 1.82), *CDKN1A* (FC_HG, 0.1_ = 0.75; FC_HG, 1.0_ = 2.17) as well as *TIMP2* (FC_HG, 0.1_ = 1.18; FC_HG, 1.0_ = 1.78) and to a lesser extent also *CEACAM1* (FC_HG, 0.1_ = 1.05; FC_HG, 1.0_ = 1.69), *CTNNA1* (FC_HG, 0.1_ = 1.21; FC_HG, 1.0_ = 1.27) and *TIMP4* (FC_HG, 0.1_ = 0.98; FC_HG, 1.0_ = 1.38, *p* = 0.093 vs FC_HG, 0.1_), while *CASP8*, *PTEN* and *TIMP1* gene expressions were less affected (FC_HG, 0.1_ = 0.89–1.02; FC_HG, 1.0_ = 0.80–1.08).

Hyperinsulinemia moderately downregulated the expression of *APC* (FC_NG, ins_ = 0.57), *CDKN1A* (FC_NG, ins_ = 0.61, *p* = 0.033 vs FC_NG, 1.0_), *CEACAM1* (FC_NG, ins_ = 0.86, *p* = 0.029 vs FC_NG, 0.1_) and *IGF1* (FC_NG, ins_ = 0.68) under normoglycemic conditions, and more remarkably of *BCL2L11* (FC_NG, ins_ = 0.51, *p* = 0.007, *p* = 0.003 vs FC_NG, 0.1_ and *p* = 0.028 vs FC_NG, 1.0_), *CASP8* (FC_NG, ins_ = 0.48, *p* = 0.099), *CDH1* (FC_NG, ins_ = 0.38, *p* = 0.001, *p* = 0.056 vs FC_NG, 0.1_), *PTEN* (FC_NG, ins_ = 0.43, *p* = 0.013), *RAC1* (FC_NG, ins_ = 0.54, *p* = 0.069 vs FC_NG, 0.1_) and *TGFB1* (FC_NG, ins_ = 0.58). Except for *TGFB1*, gene expression was lower in a hyperinsulinemic environment compared to the metformin-induced effects, which were more favorable only for *IGF1* and *RAC1* expression, while especially *BCL2L11*, *CASP8*, *CDH1* and *PTEN* expressions were negatively affected by enhanced insulin levels. The expression of the remaining genes was not affected by elevated insulin levels under normal glucose conditions (FC_NG, ins_ = 0.78–1.16).

In a hyperglycemic environment, however, insulin supplementation slightly upregulated *CDH1* (FC_HG, ins_ = 1.43), *CEACAM1* (FC_HG, ins_ = 1.41, *p* = 0.083), *IGF1* (FC_HG, ins_ = 1.28), *MMP2* (FC_HG, ins_ = 1.53), *MMP9* (FC_HG, ins_ = 1.28; *p* = 0.044 vs FC_HG, 0.1_ and *p* = 0.017 vs FC_HG, 1.0_) and *TIMP4* (FC_HG, ins_ = 1.54), but the effects were only more favorable in the case of *TIMP4* compared to a single metformin treatment. No insulin-induced effects on the expression of the remaining genes were observed during hyperglycemia (FC_HG, ins_ = 0.93–1.20).

A treatment with metformin in a hyperinsulinemic and normoglycemic environment reversed the detected downregulation that has been induced by insulin alone for the following genes: *APC* (FC_NG, 0.1+ins_ = 0.80; FC_NG, 1.0+ins_ = 0.75), *BCL2L11* (FC_NG, 0.1+ins_ = 0.82, *p* = 0.024 vs FC_NG, ins_; FC_NG, 1.0+ins_ = 0.90, *p* = 0.075 vs FC_NG, ins_), *CASP8* (FC_NG, 0.1+ins_ = 0.62; FC_NG, 1.0+ins_ = 0.57, *p* = 0.078), *CDH1* (FC_NG, 0.1+ins_ = 0.61; FC_NG, 1.0+ins_ = 0.56), *CDKN1A* (FC_NG, 0.1+ins_ = 0.74; FC_NG, 1.0+ins_ = 0.89), *CEACAM1* (FC_NG, 0.1+ins_ = 1.27; FC_NG, 1.0+ins_ = 1.15), *PTEN* (FC_NG, 0.1+ins_ = 0.70, *p* = 0.034 vs FC_NG, ins_; FC_NG, 1.0+ins_ = 0.51), *RAC1* (FC_NG, 0.1+ins_ = 0.76; FC_NG, 1.0+ins_ = 0.80), *TIMP4* (FC_NG, 0.1+ins_ = 0.90; FC_NG, 1.0+ins_ = 1.59, *p* = 0.015 vs FC_NG, ins_ and *p* = 0.028 vs FC_NG, 0.1+ins_). This attenuating metformin effect on the insulin-induced downregulation was mostly favorable, except for *RAC1*. Metformin effects on *IGF1* (FC_NG, 0.1+ins_ = 0.92; FC_NG, 1.0+ins_ = 0.58) expression were more favorable at 1.0 mmol/L in the presence of insulin compared to a single treatment with the biguanide drug. No effects on metformin-induced changes in gene expression were observed after an additional supplementation with insulin for *CTNNA1* (FC_NG, 0.1+ins_ = 1.04; FC_NG, 1.0+ins_ = 0.98), *MMP2* (FC_NG, 0.1+ins_ = 1.00; FC_NG, 1.0+ins_ = 1.61), *MMP9* (FC_NG, 0.1+ins_ = 0.89, *p* = 0.097 vs FC_NG, ins_; FC_NG, 1.0+ins_ = 0.38, *p* = 0.021 vs FC_NG, 1.0_ and *p* = 0.074 vs FC_NG, 0.1+ins_), *TGFB1* (FC_NG, 0.1+ins_ = 0.70; FC_NG, 1.0+ins_ = 0.48, *p* = 0.035), *TIMP1* (FC_NG, 0.1+ins_ = 1.10; FC_NG, 1.0+ins_ = 1.08) and *TIMP2* (FC_NG, 0.1+ins_ = 1.02; FC_NG, 1.0+ins_ = 1.23) compared to a single metformin treatment under normoglycemic conditions.

In a hyperinsulinemic and simultaneously hyperglycemic milieu, treatment with 1.0 mmol/L metformin slightly reduced gene expression of *CASP8* (FC_HG, 0.1+ins_ = 1.19; FC_HG, 1.0+ins_ = 0.80) and *CDH1* (FC_HG, 0.1+ins_ = 1.12; FC_HG, 1.0+ins_ = 1.39), while *MMP9* (FC_HG, 0.1+ins_ = 1.32; FC_HG, 1.0+ins_ = 0.49, *p* = 0.0007 vs FC_HG, 0.1+ins_ and *p* = 0.009 vs FC_HG, ins_) expression was slightly higher compared to a treatment with metformin alone, indicating a negative influence of insulin on the expression of those genes. Combination of insulin and 1.0 mmol/L metformin also enhanced the expression levels of *CEACAM1* (FC_HG, 0.1+ins_ = 1.96; FC_HG, 1.0+ins_ = 5.40, *p* = 0.060, *p* = 0.086 vs FC_HG, 0.1_ and *p* = 0.075 vs FC_HG, ins_), *TIMP4* (FC_HG, 0.1+ins_ = 1.48, *p* = 0.070 vs FC_HG, 0.1_; FC_HG, 1.0+ins_ = 3.00, *p* = 0.088) and to a lesser extent of *APC* (FC_HG, 0.1+ins_ = 1.15; FC_HG, 1.0+ins_ = 2.22, *p* = 0.058 and *p* = 0.036 vs FC_HG, ins_), *BCL2L11* (FC_HG, 0.1+ins_ = 1.03; FC_HG, 1.0+ins_ = 3.02, *p* = 0.087 vs FC_HG, ins_, *p* = 0.080 vs FC_HG, 0.1+ins_ and *p* = 0.073 vs FC_NG, 1.0+ins_), *CTNNA1* (FC_HG, 0.1+ins_ = 1.35, *p* = 0.058; FC_HG, 1.0+ins_ = 1.27) as well as *TIMP2* (FC_HG, 0.1+ins_ = 1.17; FC_HG, 1.0+ins_ = 1.95, *p* = 0.087 and *p* = 0.071 vs FC_HG, ins_), but here, the insulin-induced enhancement of gene expression was rather advantageous. Only an increased upregulation of *TIMP1* (FC_HG, 0.1+ins_ = 1.12; FC_HG, 1.0+ins_ = 1.29) after combined treatment was unfavorable under hyperglycemic conditions. Furthermore, the presence of insulin did not affect metformin-induced changes compared to single metformin supplementation of other genes (FC_HG, 0.1+ins_ = 0.91–1.20; FC_HG, 1.0+ins_ = 0.38–2.10).

Glucose-induced changes in gene expression between identically treated samples were observed, but were not significant (Fig. S1). When untreated controls under normo- and hyperglycemic conditions were compared, glucose supplementation substantially downregulated *APC* (FC_HG, control_ = 0.51), *CASP8* (FC_HG, control_ = 0.61), *CDH1* (FC_HG, control_ = 0.25), *PTEN* (FC_HG, control_ = 0.23), *TIMP4* (FC_HG, control_ = 0.21) and even significantly *CEACAM1* expression (FC_HG, control_ = 0.09, *p* = 0.016). *CEACAM1* was also remarkably downregulated by high glucose levels under any other tested condition (FC_1.0, HG vs NG_ = 0.25; FC_ins, HG vs NG_ = 0.15; FC_1.0+ins, HG vs NG_ = 0.43), particularly with 0.1 mmol/L metformin (FC_0.1, HG vs NG_ = 0.09, *p* = 0.029; FC_0.1+ins, HG vs NG_ = 0.14, *p* = 0.016). In contrast, hyperglycemia increased the levels of *CDKN1A* (FC_HG, control_ = 1.20), *MMP2* (FC_HG, control_ = 2.62), *TIMP1* (FC_HG, control_ = 1.29) and *BCL2L11* (FC_HG, control_ = 1.29) compared to untreated normoglycemic controls with the latter being affected by elevated glucose levels especially in the presence of 1.0 mmol/L metformin (FC_1.0, HG vs NG_ = 3.18, *p* = 0.096; FC_1.0+ins, HG vs NG_ = 4.34, *p* = 0.073). The remaining genes were less prone to hyperglycemia (FC_HG, control_ = 0.74–1.12).

In Ishikawa cells, *MMP10* (FC_NG, 0.1_ = 0.78; FC_NG, 1.0_ = 1.21) and *TIMP3* (FC_NG, 0.1_ = 0.67, *p* = 0.069; FC_NG, 1.0_ = 1.17) were downregulated after treatment with 0,1 mmol/L metformin under normoglycemic conditions, but were upregulated at 1.0 mmol/L, while *TIMP4* expression (FC_NG, 0.1_ = 0.76; FC_NG, 1.0_ = 1.00) remained unchanged at the high metformin concentration. *BCL2L11* (FC_NG, 0.1_ = 0.67, *p* = 0.053; FC_NG, 1.0_ = 0.71) and *MMP2* (FC_NG, 0.1_ = 0.65; FC_NG, 1.0_ = 0.69) expressions were downregulated irrespective of the applied metformin concentration. The remaining genes were all downregulated by metformin in a concentration-dependent manner: *CASP8* (FC_NG, 0.1_ = 0.72; FC_NG, 1.0_ = 0.60), *CDH1* (FC_NG, 0.1_ = 0.55; FC_NG, 1.0_ = 0.44, *p* = 0.095), *CDKN1A* (FC_NG, 0.1_ = 0.66; FC_NG, 1.0_ = 0.59), *CTNNA1* (FC_NG, 0.1_ = 0.86; FC_NG, 1.0_ = 0.70), *IGF1* (FC_NG, 0.1_ = 0.60; FC_NG, 1.0_ = 0.49), *MMP9* (FC_NG, 0.1_ = 0.92; FC_NG, 1.0_ = 0.52, *p* = 0.058), *PTEN* (FC_NG, 0.1_ = 0.80; FC_NG, 1.0_ = 0.60, *p* = 0.058), *RAC1* (FC_NG, 0.1_ = 0.74; FC_NG, 1.0_ = 0.49, *p* = 0.071), *TIMP1* (FC_NG, 0.1_ = 0.87; FC_NG, 1.0_ = 0.83) and *TIMP2* (FC_NG, 0.1_ = 0.76; FC_NG, 1.0_ = 0.65, *p* = 0.054). A significant downregulation was observed for *APC* (FC_NG, 0.1_ = 0.59; FC_NG, 1.0_ = 0.43, *p* = 0.030) and *TGFB1* (FC_NG, 0.1_ = 0.68; FC_NG, 1.0_ = 0.57, *p* = 0.042).

In contrast, a slight concentration-dependent upregulation was detected for *CDKN1A* (FC_HG, 0.1_ = 0.99; FC_HG, 1.0_ = 1.64), *PTEN* (FC_HG, 0.1_ = 1.07; FC_HG, 1.0_ = 1.21), *RAC1* (FC_HG, 0.1_ = 1.04; FC_HG, 1.0_ = 1.58), *TGFB1* (FC_HG, 0.1_ = 1.07; FC_HG, 1.0_ = 1.31), *TIMP1* (FC_HG, 0.1_ = 1.09; FC_HG, 1.0_ = 1.24), *TIMP2* (FC_HG, 0.1_ = 1.06; FC_HG, 1.0_ = 1.25), and *TIMP4* (FC_HG, 0.1_ = 1.04; FC_HG, 1.0_ = 1.46) under hyperglycemic conditions. *MMP2* (FC_HG, 0.1_ = 0.79; FC_HG, 1.0_ = 0.81) and *MMP10* (FC_HG, 0.1_ = 0.89; FC_HG, 1.0_ = 0.68) genes were downregulated at both metformin concentrations. Opposing metformin effects were detected for the following genes: *APC* (FC_HG, 0.1_ = 0.89; FC_HG, 1.0_ = 1.31), *COL1A1* (FC_HG, 0.1_ = 0.81; FC_HG, 1.0_ = 1.25) and *TIMP3* (FC_HG, 0.1_ = 0.81; FC_HG, 1.0_ = 1.09, *p* = 0.007 vs FC_HG, 0.1_) were downregulated after treatment with 0.1 mmol/L metformin, but upregulated at 1.0 mmol/L, while the expression of all remaining genes has not been changed (FC_HG, 0.1_ = 0.89–1.15; FC_HG, 1.0_ = 0.97–1.12).

The expression of most tested genes was downregulated by insulin under normoglycemic conditions, with most prominent changes for *APC* (FC_NG, ins_ = 0.51), *BCL2L11* (FC_NG, ins_ = 0.63), *CDKN1A* (FC_NG, ins_ = 0.49, *p* = 0.081), *CASP8* (FC_NG, ins_ = 0.55), *CDH1* (FC_NG, ins_ = 0.43), *COL1A1* (FC_NG, ins_ = 0.48), *IGF1* (FC_NG, ins_ = 0.52), *PTEN* (FC_NG, ins_ = 0.66), *TGFB1* (FC_NG, ins_ = 0.58, *p* = 0.066), *TIMP2* (FC_NG, ins_ = 0.68, *p* = 0.065) and even significant downregulation for *TIMP3* (FC_NG, ins_ = 0.62, *p* = 0.001 and *p* = 0.005 vs FC_NG, 1.0_). The remaining genes were only minimally affected by hyperinsulinemia (FC_NG, ins_ = 0.77–0.92).

Under hyperglycemic conditions, insulin supplementation did not induce remarkable changes in the expression of any analyzed gene (FC_HG, ins_ = 0.90–1.35).

In a combined treatment, metformin reversed the insulin-induced downregulation of *APC* (FC_NG, 0.1+ins_ = 0.57; FC_NG, 1.0+ins_ = 0.59), *BCL2L11* (FC_NG, 0.1+ins_ = 0.72; FC_NG, 1.0+ins_ = 0.90, *p* = 0.016 vs FC_NG, ins_), *CASP8* (FC_NG, 0.1+ins_ = 0.66; FC_NG, 1.0+ins_ = 0.75), *CDH1* (FC_NG, 0.1+ins_ = 0.52; FC_NG, 1.0+ins_ = 0.56), *CDKN1A* (FC_NG, 0.1+ins_ = 0.61; FC_NG, 1.0+ins_ = 0.78 *p* = 0.011 vs FC_NG, ins_), *COL1A1* (FC_NG, 0.1+ins_ = 0.78; FC_NG, 1.0+ins_ = 0.77), *PTEN* (FC_NG, 0.1+ins_ = 0.73; FC_NG, 1.0+ins_ = 0.89), *TGFB1* (FC_NG, 0.1+ins_ = 0.67; FC_NG, 1.0+ins_ = 0.69) and *TIMP4* (FC_NG, 0.1+ins_ = 0.90; FC_NG, 1.0+ins_ = 1.06) during normoglycemia, which are desired effects for all genes except *COL1A1* and *TGFB1*, where lower levels are more favorable. No differences appeared in comparison to gene expression after single metformin treatment for *CTNNA1* (FC_NG, 0.1+ins_ = 0.92; FC_NG, 1.0+ins_ = 0.70, *p* = 0.071 and *p* = 0.074 vs FC_NG, ins_), *IGF1* (FC_NG, 0.1+ins_ = 0.55; FC_NG, 1.0+ins_ = 0.64), *MMP2* (FC_NG, 0.1+ins_ = 0.63; FC_NG, 1.0+ins_ = 0.65), *MMP9* (FC_NG, 0.1+ins_ = 0.75; FC_NG, 1.0+ins_ = 0.69), *MMP10* (FC_NG, 0.1+ins_ = 0.64; FC_NG, 1.0+ins_ = 1.20), *RAC1* (FC_NG, 0.1+ins_ = 0.67; FC_NG, 1.0+ins_ = 0.61), *TIMP1* (FC_NG, 0.1+ins_ = 0.97; FC_NG, 1.0+ins_ = 0.83), *TIMP2* (FC_NG, 0.1+ins_ = 0.82; FC_NG, 1.0+ins_ = 0.68, *p* = 0.01) and *TIMP3* (FC_NG, 0.1+ins_ = 0.71; FC_NG, 1.0+ins_ = 1.27, *p* = 0.078 vs FC_NG, 1.0_).

In a hyperglycemic environment, additional insulin supplementation reduced or even reversed the metformin-induced upregulation of *APC* (FC_HG, 0.1+ins_ = 0.87; FC_HG, 1.0+ins_ = 0.83), *CDKN1A* (FC_HG, 0.1+ins_ = 0.99; FC_HG, 1.0+ins_ = 1.17), *COL1A1* (FC_HG, 0.1+ins_ = 0.73; FC_HG, 1.0+ins_ = 0.82), *CTNNA1* (FC_HG, 0.1+ins_ = 0.87; FC_HG, 1.0+ins_ = 0.87), *MMP9* (FC_HG, 0.1+ins_ = 1.15; FC_HG, 1.0+ins_ = 1.12), *TIMP1* (FC_HG, 0.1+ins_ = 0.94; FC_HG, 1.0+ins_ = 0.86), *TIMP2* (FC_HG, 0.1+ins_ = 0.89; FC_HG, 1.0+ins_ = 0.92), *TIMP3* (FC_HG, 0.1+ins_ = 0.91; FC_HG, 1.0+ins_ = 0.81) and *TIMP4* (FC_HG, 0.1+ins_ = 0.95; FC_HG, 1.0+ins_ = 1.03). For the *MMP2*, insulin enhanced the metformin-mediated downregulation, especially when combined with 1.0 mmol/L (FC_HG, 0.1+ins_ = 0.68; FC_HG, 1.0+ins_ = 0.58). No effects on the other genes were observed after combination compared to single metformin treatment (FC_HG, 0.1+ins_ = 0.77–1.07; FC_HG, 1.0+ins_ = 0.67–1.27).

Glucose-induced changes in gene expression were observed, but were not significant (Fig. S1). Comparing the untreated controls under normo- and hyperglycemic conditions, glucose supplementation downregulated *APC* (FC_HG, control_ = 0.65), *BCL2L11* (FC_HG, control_ = 0.63), *CASP8* (FC_HG, control_ = 0.66), *CDH1* (FC_HG, control_ = 0.61), *CDKN1A* (FC_HG, control_ = 0.62), *MMP2* (FC_HG, control_ = 0.68), *MMP10* (FC_HG, control_ = 0.65) as well as *TGFB1* (FC_HG, control_ = 0.65), while the remaining genes were less affected (FC_HG, control_ = 0.71–1.06).

Changes in the Expression of Selected Proteins after Metformin Treatment under Different Metabolic Conditions in Western Blot Analysis.

**Fig. 3:**
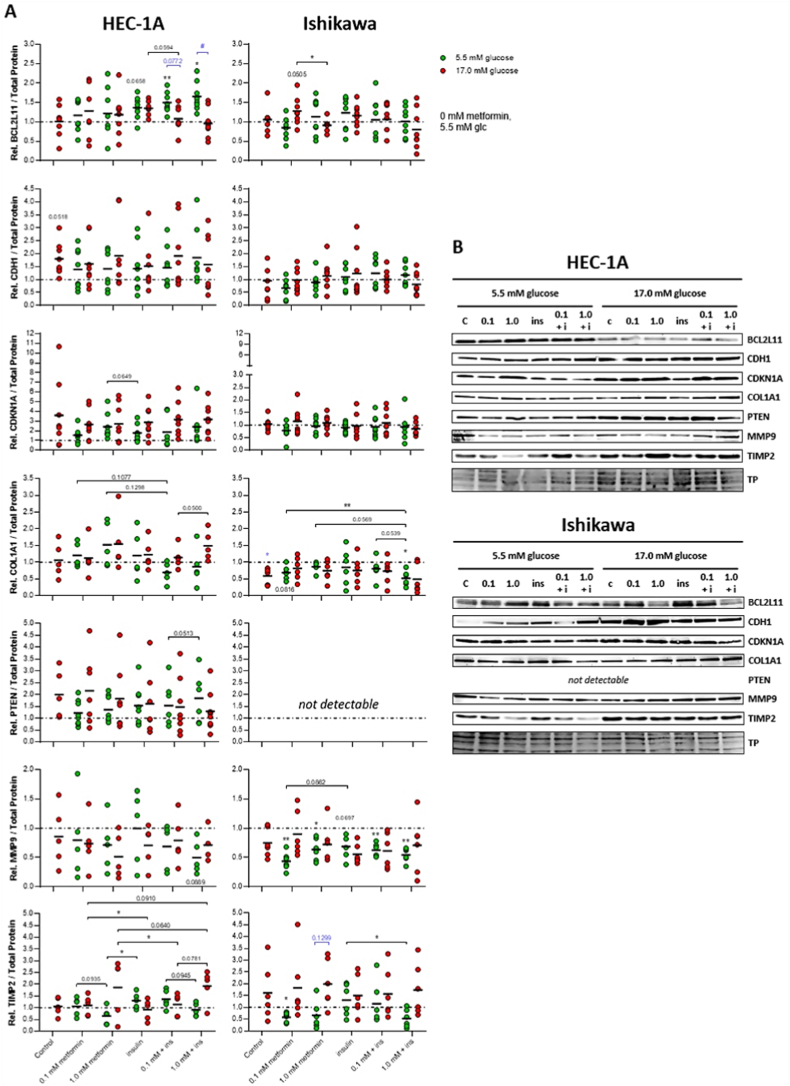
Changes in the expression of selected proteins after treatment of HEC-1A and Ishikawa cells with metformin, insulin, or a combination of both substances for 7 d. (**a**) Cells were treated with 0.1 and 1.0 mmol/L metformin, 100 ng/mL insulin or a combination of metformin and insulin under normo- (5.5 mmol/L glucose, green) or hyperglycemic conditions (17.0 mmol/L glucose, red) for 7 d and western blotting was performed afterwards. Semi-quantitative, densitometric analysis was carried out in order to determine the relative normalized protein expression (fold-change). Untreated cells cultivated under normoglycemic conditions served as the reference group (fold-change was set to 1.0 for this sample as indicated by a dotted line). Data presented as dot plots with arithmetic means of at least three independent experiments; n = 5–7. Significant differences were determined with a mixed effects model analysis and subsequent Tukey's (analysis of metformin and insulin effects under identical glucose conditions) or Šídák's (analysis of glucose effects between identical treatments) multiple comparison *post-hoc* tests; *p ≤ 0.05, **p ≤ 0.01 (metformin effect, black); ^#^*p* ≤ 0.05 (glucose effect, blue); *p* < 0.15 additionally displayed as values. (**b**) Representative blots are shown with transferred total protein (TP) used for normalization; BCL2L11: 23 kDa, CDH1: 135 kDa, CDKN1A: 21 kDa, COL1A1: 110 kDa, PTEN: 54 kDa, MMP9: 63 kDa, TIMP2: 22 kDa (Figure S3).

After gene expression analysis, 7 genes were selected for analysis on protein level by western blotting (Fig. 3). PTEN could not be detected in the premenopausal Ishikawa cells, but this protein is known to be absent in this cell line [40,41]. Unless stated otherwise, *p* values refer to the untreated normoglycemic control sample; *p* values < 0.1 were mentioned in the text.

In HEC-1A cells, metformin slightly upregulated BCL2L11 (FC_NG, 0.1_ = 1.17; FC_NG, 1.0_ = 1.22), CDH1 (FC_NG, 0.1_ = 1.39; FC_NG, 1.0_ = 1.41) and PTEN levels (FC_NG, 0.1_ = 1.22; FC_NG, 1.0_ = 1.36) independent of the concentration under normoglycemic conditions, whereas a concentration-dependent and more substantial increase was observed for CDKN1A (FC_NG, 0.1_ = 1.55; FC_NG, 1.0_ = 2.41) and COL1A1 expression (FC_NG, 0.1_ = 1.20; FC_NG, 1.0_ = 1.52). MMP9 (FC_NG, 0.1_ = 0.80; FC_NG, 1.0_ = 0.71) and TIMP2 levels (FC_NG, 0.1_ = 1.06; FC_NG, 1.0_ = 0.65, *p* = 0.094 vs FC_NG, 0.1_) were lowered in the presence of metformin at 1.0 mmol/L.

For hyperglycemic conditions, it has to be noted that the increase in glucose induced remarkable elevations of CDH1 (FC_HG, control_ = 1.80), CDKN1A (FC_HG, control_ = 3.60) and PTEN levels (FC_HG, control_ = 2.00) in the untreated hyperglycemic control sample (Fig. S2), whereas no glucose-mediated effects on the expression of the remaining analyzed proteins were detected (FC_HG, control_ = 0.86–1.17). Keeping that in mind, effects of metformin treatment during hyperglycemia were similar to the effects observed in a normoglycemic milieu for BCL2L11 (FC_HG, 0.1_ = 1.28; FC_HG, 1.0_ = 1.19), COL1A1 (FC_HG, 0.1_ = 1.12; FC_HG, 1.0_ = 1.55) and MMP9 (FC_HG, 0.1_ = 0.74; FC_HG, 1.0_ = 0.51), whereas CDH1 (FC_HG, 0.1_ = 1.60; FC_HG, 1.0_ = 1.92) and PTEN (FC_HG, 0.1_ = 2.16; FC_HG, 1.0_ = 1.82) levels were not affected by metformin under the influence of hyperglycemia. Opposing metformin effects were even detected for CDKN1A and TIMP2 in a high glucose environment when compared to the respective untreated hyperglycemic control samples (CDKN1A: FC_HG, control_ = 3.60; TIMP2: FC_HG, control_ = 1.06), where CDKN1A (FC_HG, 0.1_ = 2.66; FC_HG, 1.0_ = 2.72) was now slightly downregulated after metformin supplementation and TIMP2 expression was now upregulated (FC_HG, 0.1_ = 1.10; FC_HG, 1.0_ = 1.86).

Insulin supplementation led to an increased expression of BCL2L11 (FC_NG, ins_ = 1.22, *p* = 0.066), CDKN1A (FC_NG, ins_ = 1.77, *p* = 0.065 vs FC_NG, 1.0_) and TIMP2 (FC_NG, ins_ = 1.31, *p* = 0.032 vs FC_NG, 1.0_) as well as all other selected proteins under normoglycemic conditions (FC_NG, ins_ = 1.20–1.53), except MMP9, which remained unchanged (FC_NG, ins_ = 1.00).

In contrast, CDH1 (FC_HG, ins_ = 1.52), CDKN1A (FC_HG, ins_ = 2.87), PTEN (FC_HG, ins_ = 1.61) and MMP9 (FC_HG, ins_ = 0.71) were slightly downregulated during hyperinsulinemia under the influence of a hyperglycemic environment, when compared to the respective untreated hyperglycemic control samples. TIMP2 levels were not affected, but significantly lower than after treatment with 1.0 mmol/L metformin (FC_HG, ins_ = 0.92, *p* = 0.043 vs FC_HG, 0.1_, *p* = 0.036 vs FC_HG, 0.1+ins_ and *p* = 0.064 vs FC_HG, 1.0+ins_). BCL2L11 (FC_HG, ins_ = 1.19) and COL1A1 (FC_HG, ins_ = 1.23) expression were increased after insulin administration independent of glucose in the medium.

A combined treatment with metformin and insulin had remarkable positive effects on BCL2L11 expression. In the presence of hyperinsulinemia, metformin further enhanced the expression of BCL2L11 in a concentration-dependent manner at 5.5 mmol/L glucose (FC_NG, 0.1+ins_ = 1.50, *p* = 0.009; FC_NG, 1.0+ins_ = 1.66, *p* = 0.012), but reversed the upregulation that was observed after treatment with metformin and particularly insulin alone at 17.0 mmol/L glucose (FC_HG, 0.1+ins_ = 1.08, *p* = 0.077 vs FC_NG, 0.1_ and *p* = 0.059 vs FC_HG, ins_; FC_HG, 1.0+ins_ = 0.96, *p* = 0.014 vs FC_NG, 1.0_), indicating an unfavorable influence of glucose during the combined treatment.

Favorable elevated amounts of CDH1 (FC_NG, 0.1+ins_ = 1.49; FC_NG, 1.0+ins_ = 1.85), PTEN (FC_NG, 0.1+ins_ = 1.53; FC_NG, 1.0+ins_ = 1.84, *p* = 0.051 vs FC_NG, 1.0+ins_) and TIMP2 (FC_NG, 0.1+ins_ = 1.36; FC_NG, 1.0+ins_ = 0.91, *p* = 0.095 vs FC_NG, 0.1+ins_) were also detected after combination under normal glucose levels when compared to single treatments, but in case of CDH1 and PTEN only at 1.0 mmol/L metformin. MMP9 expression (FC_NG, 0.1+ins_ = 0.69; FC_NG, 1.0+ins_ = 0.50, *p* = 0.089) was further decreased when 1.0 mmol/L metformin was administered under the influence of hyperinsulinemia in a normal glucose environment, indicating a supportive anti-metastatic effect. CDKN1A expression (FC_NG, 0.1+ins_ = 1.86; FC_NG, 1.0+ins_ = 2.41) did not change after a combined treatment. COL1A1 expression (FC_NG, 0.1+ins_ = 0.69; FC_NG, 1.0+ins_ = 0.87) was even regulated in the opposite direction by metformin in the presence of insulin, which was more favorable.

When elevated concentrations of glucose were applied, expression of CDH1 (FC_HG, 0.1+ins_ = 1.92; FC_HG, 1.0+ins_ = 1.58), CDKN1A (FC_HG, 0.1+ins_ = 3.16; FC_HG, 1.0+ins_ = 3.17), COL1A1 (FC_HG, 0.1+ins_ = 1.14; FC_HG, 1.0+ins_ = 1.49, *p* = 0.050 vs FC_HG, 0.1+ins_) and TIMP2 (FC_HG, 0.1+ins_ = 1.14; FC_HG, 1.0+ins_ = 1.93, *p* = 0.078 vs FC_HG, 0.1+ins_) did not vary between single and combined treatments, while BCL2L11 (FC_HG, 0.1+ins_ = 1.08, *p* = 0.059 vs FC_HG, ins_; FC_HG, 1.0+ins_ = 0.96) and PTEN levels (FC_HG, 0.1+ins_ = 1.61; FC_HG, 1.0+ins_ = 1.47) were reduced after combined treatment, indicating an inhibiting insulin effect on the anti-tumor effects of metformin. In the case of MMP9 (FC_HG, 0.1+ins_ = 0.79; FC_HG, 1.0+ins_ = 0.71), protein expression was normalized to levels similar to the untreated control, which was rather disadvantageous in terms of inhibition of metastasis.

An overview of the metformin-induced changes on the expression of the 7 selected key molecular targets on gene and protein level is provided in Table 3 for the postmenopausal HEC-1A cell line (see Graphical Abstract for a schematic overview).

**Table 3:** Metformin-induced changes on the expression of 7 key molecular targets on gene and protein level under normo- and hyperglycemic conditions as well as under the influence of hyperinsulinemia for the postmenopausal HEC-1A cell line.

Molecular Target (favorable regulation)	Metformin-induced Change in Expression (*Gene*/Protein Level)			
*Gene*/Protein	Normoglycemia	+ Insulin Effect	Hyperglycemia	+ Insulin Effect
*BCL2L11*/BCL2L11 (↑)	-/↑	fav./fav.	↑/↑	fav./unfav.
*CDH1*/CDH1 (↑)	↓/↑	fav./fav.	↑/-	unfav./-
*CDKN1A*/CDKN1A (↑)	-/↑	fav./-	↑/↓	-/-
*COL1A1*/COL1A1 (↓)	n.d./↑	n.d./fav.	n.d./↑	n.d./-
*MMP9*/MMP9 (↓)	↓/↓	-/fav.	↓/↓	unfav./unfav.
*PTEN*/PTEN (↑)	↓/↑	fav./fav.	-/↓	-/unfav.
*TIMP2*/TIMP2 (↑)	-/↓	-/fav.	↑/↑	fav./-

↑ – upregulation; ↓ – downregulation; n.d. - not detectable; fav. – favorable; unfav. – unfavorable; - – no effect.

In Ishikawa cells, metformin administration had a slightly downregulating effect on the expression of BCL2L11 (FC_NG, 0.1_ = 0.85; FC_NG, 1.0_ = 1.13) and CDKN1A (FC_NG, 0.1_ = 0.78; FC_NG, 1.0_ = 0.96) at 0.1 mmol/L under normal glucose concentrations, which was more remarkable for CDH1 (FC_NG, 0.1_ = 0.67; FC_NG, 1.0_ = 0.89), COL1A1 (FC_NG, 0.1_ = 0.69, *p* = 0.082; FC_NG, 1.0_ = 0.87) and even significant for TIMP2 (FC_NG, 0.1_ = 0.58, *p* = 0.024; FC_NG, 1.0_ = 0.66), while MMP9 levels (FC_NG, 0.1_ = 0.44, *p* = 0.002; FC_NG, 1.0_ = 0.64, *p* = 0.035) were significantly lowered by metformin at both concentrations.

Under hyperglycemic conditions, upregulation appeared only for BCL2L11 (FC_HG, 0.1_ = 1.28, *p* = 0.051; FC_HG, 1.0_ = 0.91, *p* = 0.037 vs FC_HG, 0.1_), COL1A1 (FC_HG, 0.1_ = 0.82; FC_HG, 1.0_ = 0.75) and MMP9 (FC_HG, 0.1_ = 0.90; FC_HG, 1.0_ = 0.72) in particular at 0.1 mmol/L in comparison to untreated cells in a hyperglycemic environment, which was in contrast to the effects observed in a normoglycemic environment at this concentration. It has to be noted that elevated glucose levels *per se* had a slightly increasing effect on the expression of TIMP2 (FC_HG, control_ = 1.62), while COL1A1 (FC_HG, control_ = 0.59, *p* = 0.041) and MMP9 levels (FC_HG, control_ = 0.75) were substantially downregulated (Fig. S2). All other proteins did not show glucose-induced alterations of expression levels between untreated control samples (FC_HG, control_ = 0.96–1.06). Under this premise, metformin also caused opposing effects on the expression of CDH1 (FC_HG, 0.1_ = 0.98; FC_HG, 1.0_ = 1.14), CDKN1A (FC_HG, 0.1_ = 1.15; FC_HG, 1.0_ = 1.08) and TIMP2 (FC_HG, 0.1_ = 1.83; FC_HG, 1.0_ = 1.99) at one of the tested concentrations.

No insulin effects on the expression of CDH1, CDKN1A and COL1A1 were observed under normoglycemia (FC_NG, ins_ = 0.85–1.10). BCL2L11 (FC_NG, ins_ = 1.23) and TIMP2 levels (FC_NG, ins_ = 1.31) were slightly increased, whereas insulin supplementation alone had a decreasing effect on the expression of MMP9 (FC_NG, ins_ = 0.64, *p* = 0.070 and *p* = 0.089 vs FC_NG, 0.1_).

Under hyperglycemic conditions, hyperinsulinemia slightly upregulated CDH1 (FC_HG, ins_ = 1.23) and COL1A1 expression (FC_HG, ins_ = 0.75) compared to the respective untreated hyperglycemic controls, whereas MMP9 levels (FC_HG, ins_ = 0.56) were downregulated. BCL2L11 (FC_HG, ins_ = 1.16), CDKN1A (FC_HG, ins_ = 0.97) and TIMP2 levels (FC_HG, ins_ = 1.50) were not affected by hyperinsulinemia.

In the presence of insulin, metformin opposed CDH1 expression (FC_NG, 0.1+ins_ = 1.24; FC_NG, 1.0+ins_ = 1.17) irrespective of the applied metformin concentration in a normoglycemic environment, which was advantageous in terms of anti-proliferative effects. Downregulation of MMP9 (FC_NG, 0.1+ins_ = 0.63, *p* = 0.003; FC_NG, 1.0+ins_ = 0.54, *p* = 0.002) and COL1A1 levels (FC_NG, 0.1+ins_ = 0.80; FC_NG, 1.0+ins_ = 0.52, *p* = 0.021, *p* = 0.054 vs FC_NG, 0.1+ins_ and *p* = 0.057 vs FC_NG, 1.0_) was reduced at the low metformin concentration under the influence of hyperinsulinemia, but was further increased when 1.0 mmol/L metformin was combined with insulin. Furthermore, a combined treatment suppressed the insulin-induced upregulation of TIMP2 (FC_NG, 0.1+ins_ = 1.15; FC_NG, 1.0+ins_ = 0.52, *p* = 0.034 vs FC_NG, ins_). In case of COL1A1, MMP9 and TIMP2, insulin supplementation led to a more favorable outcome when combined at high concentration. BCL2L11 and CDKN1A levels remained unchanged compared to the respective single treatments (FC_NG, 0.1+ins_ = 0.93–1.06; FC_NG, 1.0+ins_ = 0.93–1.02).

Under hyperglycemic conditions, downregulation of BCL2L11 (FC_HG, 0.1+ins_ = 1.07; FC_HG, 1.0+ins_ = 0.80) was enhanced at 1.0 mmol/L, while CDH1 (FC_HG, 0.1+ins_ = 1.00; FC_HG, 1.0+ins_ = 0.81) and COL1A1 (FC_HG, 0.1+ins_ = 0.73; FC_HG, 1.0+ins_ = 0.49) were now also downregulated, which was in contrast to identical treatments at normal glucose levels, but advantageous in case of COL1A1. MMP9 expression (FC_HG, 0.1+ins_ = 0.61; FC_HG, 1.0+ins_ = 0.71) was opposed when 0.1 mmol/L metformin was combined with insulin, leading to a more desirable effect. CDKN1A (FC_HG, 0.1+ins_ = 1.09; FC_HG, 1.0+ins_ = 0.85) as well as TIMP2 levels (FC_HG, 0.1+ins_ = 1.57; FC_HG, 1.0+ins_ = 1.73) were similar to levels observed after single compound administration.

An overview of the metformin-induced changes on the expression of the 7 selected key molecular targets on gene and protein level is provided in Table 4 for the premenopausal Ishikawa cell lines (see Graphical Abstract for a schematic overview).

**Table 4:** Metformin-induced changes on the expression of 7 key molecular targets on gene and protein level under normo- and hyperglycemic conditions as well as under the influence of hyperinsulinemia for the premenopausal Ishikawa cell line.

Molecular Target (favorable regulation)	Metformin-induced Change in Expression (*Gene*/Protein Level)			
*Gene*/Protein	Normoglycemia	+ Insulin Effect	Hyperglycemia	+ Insulin Effect
*BCL2L11*/BCL2L11 (↑)	↓/↑	fav./-	-/↑	-/unfav.
*CDH1*/CDH1 (↑)	↓/-	fav./fav.	-/-	-/unfav.
*CDKN1A*/CDKN1A (↑)	↓/-	fav./-	↑/-	unfav./-
*COL1A1*/COL1A1 (↓)	↑/↓	unfav./fav.	↑/↑	fav./fav.
*MMP9*/MMP9 (↓)	↓/↓	-/fav.	-/↑	fav./fav.
*PTEN*/PTEN (↑)	↓/n.d.	fav./n.d.	-/n.d.	-/n.d.
*TIMP2*/TIMP2 (↑)	↓/↓	-/fav.	↑/↑	unfav./-

↑ – upregulation; ↓ – downregulation; n.d. - not detectable; fav. – favorable; unfav. – unfavorable; - – no effect.

## 4 Discussion

Transcriptomic analysis of EC cells was used as a screening tool in order to identify target genes that are substantially regulated by metformin administration. The screening was performed with a single selected condition, i.e. metformin treatment at 1.0 mmol/L under hyperglycemic conditions against untreated control cells. This condition was selected in order to incorporate both potential influencing factors that might affect gene expression in our *in vitro* model, namely the insulin-sensitizing and glucose-regulating drug metformin on the one hand as well as glucose itself on the other hand. The selected metformin concentration led to the inhibition of proliferation, migration and clonogenicity in both cell lines under similar experimental conditions in an *in vitro* study recently published by our group [12]. As anti-tumor effects on EC cells were the scope of our investigation, the genes used in the screening were all related to human cancer and metastasis. The following 19 potential target genes were selected for a subsequent gene expression analysis including further treatment conditions: *APC*, *BCL2L11*, *CASP8*, *CDH1*, *CDKN1A*, *CEACAM1*, *COL1A1*, *CTNNA1*, *IGF1*, *MMP2/9/10*, *PTEN*, *RAC1*, *TGFB1*, *TIMP1/2/3/4*. All genes were ≥ 2-fold up- or downregulated in transcriptomic analysis and the regulations were considered favorable. The following 7 proteins were additionally analyzed for changes in protein expression: BCL2L11, CDH1, CDKN1A, COL1A1, PTEN, MMP9, TIMP2.

Subsequent target gene expression analysis included further treatment conditions to allow for a more detailed evaluation of the influences on metformin-induced effects and to identify optimal conditions for the drug's anti-tumor effects to occur. Metformin-induced effects on the expression of 19 selected genes and 7 proteins are discussed below taking into consideration the influences of various treatment conditions.

The APC protein acts as a tumor suppressor and downregulation or loss of function of the *APC* gene are associated with EC [42,43]. In both cell lines, favorable upregulation of *APC* expression was observed at 1.0 mmol/L metformin only under hyperglycemic conditions, with the effects being more prominent and independent of the presence of insulin in HEC-1A cells. On the other hand, unfavorable downregulation occurred under normal glucose levels after metformin treatment and particularly during hyperinsulinemia, indicating an increased risk for EC promotion especially in the premenopausal Ishikawa model. In patients, however, genetic aberrations leading to nonfunctional *APC* had no influence on recurrence and metastasis in stage I EC [43] and therefore the predictive value of altered *APC* expression is at least questionable.

CTNNA1 has been shown to interact with APC [44] and inhibits cell proliferation, invasion and epithelial-mesenchymal transition (EMT) [45]. While *CTNNA1* expression was negatively affected by metformin and a combined treatment together with insulin under normoglycemia in Ishikawa cells, desired upregulation was induced under any tested condition under the influence of high glucose levels in HEC-1A cells. Similar to the regulation of *APC*, our results suggest a positive influence of glucose on the metformin-induced regulation of *CTNNA1*.

*CTNNA1*, *CTNNB1* and *CDH1* expressions were found to be suppressed in EC as well as other gynecological cancers, and further decreased with advanced invasion, contributing to cell-to-cell junctional dysfunction [46,47]. Loss of CDH1 (*E*-cadherin) and CTNNB1 expressions resulted in increased cell motility and advanced cancer stages [48,49] and are associated with EMT, a key event during EC development [50]. Inhibition of EMT with up to 5.0 mmol/L metformin for 24–48 h was shown to act via CDH1 upregulation in Ishikawa as well as HEC-50 cells *in vitro* and was also detected in tumors of diabetic EC patients [51,52]. In our study, *CDH1* transcript expression was not changed in Ishikawa cells during hyperglycemia, but metformin-mediated upregulation occurred in the postmenopausal HEC-1A cell line at 1.0 mmol/L. Unfavorable *CDH1* downregulation was detected in both cell lines under normoglycemic conditions, once again indicating a positive impact of increased glucose levels on metformin-induced expression changes of cancer-related genes. However, deviating results were obtained for CDH1 expression on protein level. In Ishikawa cells, CDH1 levels were not substantially changed under any tested condition, whereas in HEC-1A cells, upregulating metformin and insulin effects occurred exclusively under the influence of normal glucose levels. Conclusively, no positive impact of a high glucose environment on metformin-induced CDH1-mediated metastasis inhibition was observed. However, it has to be noted that CDH1 expression was higher in the untreated hyperglycemic control compared to its normoglycemic counterpart, suggesting a positive effect of glucose *per se*.

TGF-β1 represses CDH1 as well as CTNNA1 expression [53] and is known as the primary inducer of EMT, but also plays an essential role in reproductive physiology [54,55]. TGF-β1 stimulated migration, invasion and growth in endometrial HEC-1A, HEC-1B, KLE and Ishikawa cells *in vitro* [[56], [57], [58]]. In our study, *TGFB1* transcripts were downregulated by the biguanide and also by insulin or a combined treatment in both cell lines in a normoglycemic environment. Our results suggest the involvement of the TGF-β pathway in the anti-metastatic mechanism of metformin, which has been observed before [59,60].

The tumor suppressor PTEN is connected to WNT/CDKN1B signaling via regulation of the PI3K/AKT pathway and is also involved in morphogenesis and growth arrest by interacting with CDH1 [61,62]. PTEN is mutated or lost in a large proportion of tumor entities and *PTEN* loss is also frequent in EC [41,63]. *PTEN* upregulation suppresses proliferation and inhibits invasion [64,65] and was shown to be induced by treatment of the Ishikawa cell line with 0.06 mmol/L metformin after 24 h in a study by Pabona and colleagues [66]. In the present study, *PTEN* levels were downregulated by metformin and insulin supplementation in Ishikawa and even more substantially in HEC-1A cells under normoglycemia. In contrast, unfavorable *PTEN* downregulation was prevented in both cell lines in a hyperglycemic environment. On protein level, PTEN could not be detected in the Ishikawa cell line, which is in accordance with known characteristics of the cell line [40,41]. In HEC-1A cells, however, PTEN expression was upregulated by metformin especially when combined with insulin in a normoglycemic environment, indicating an anti-proliferative effect, which was not predictable on transcript level. A hyperglycemic environment generally upregulated PTEN expression, but the effects of metformin were opposed, suggesting a metformin-induced negative regulation of PTEN with high glucose levels. Ambiguous results were obtained in other studies, where PTEN downregulation was observed after treatment with 10.0 mmol/L for 24 h in 3T3-L1 preadipocyte cells [67] and with 1.0–10.0 mmol/L for 24–72 h in Huh7.5 hepatocytes [68], whereas upregulation occurred with 27.0 mmol/L after 24 h in hepatocellular carcinoma cell lines MHCC97H and Hep3B [69].

PTEN expression not only affects cellular proliferation and growth, but the tumor suppressor also inhibits migration via suppressing effects on various MMPs, including MMP2 and MMP9 [[69], [70], [71]]. *MMP2* and *MMP9* downregulation and protein inhibition are desired effects, as their expression levels are closely related to invasion and metastasis [72,73], which also applies for endometrial tumors [[74], [75], [76]]. In a study with the breast cancer cell line MCF-7, metformin displayed inhibiting effects on MMP9 expression at 5.0 and 10.0 mmol/L after 24 h, whereas MMP2 levels were not affected [77]. In the present study, *MMP2* expression was slightly downregulated in Ishikawa cells irrespective of the applied treatment condition under normoglycemic conditions, while metformin upregulated *MMP2* levels in HEC-1A cells. However, *MMP9* expression was downregulated after metformin administration in Ishikawa and particularly in HEC-1A cells in a normoglycemic environment and in the latter also in the presence of elevated glucose concentrations. Favorable metformin-induced downregulation of MMP9 was confirmed on protein level for both cell lines, suggesting MMP9 as an actively involved protein in the anti-metastatic mechanism of the drug. A negative influence of glucose on the metformin-mediated effects on MMP9 expression was only observed in the Ishikawa premenopausal model. For *MMP10*, another metastasis-promoting matrix metalloproteinase [78,79], downregulation was achieved in Ishikawa cells with 1.0 mmol/L metformin during hyperglycemia, while 0.1 mmol/L metformin was more favorable under normoglycemic conditions, particularly in combination with insulin; the *MMP10* transcript could not be detected in HEC-1A cells.

Although MMP2 and MMP9 belong to the group of gelatin binding MMPs, collagens serve as substrates for both proteinases as well. In a recent study by our group, HEC-1A cells were treated with 0.5 mmol/L metformin for 7 d and cellular lysates were analyzed for proteomic changes. Among other proteins, COL1A1 was significantly downregulated by metformin [34]. COL1A1, together with COL1A2, forms type I collagen, which is the major component of the extracellular matrix (ECM) in connective tissues and *COL1A1* displayed tumor-promoting effects in various cancer cells [[80], [81], [82]]. However, in EC patients, the survival outcome was better with high expression of *COL1A1* transcripts, whereas COL1A1 protein expression appeared to be increased in EC tissues versus normal endometrium [83]. In the present study, *COL1A1* was not detectable in HEC-1A cells and was negatively regulated in Ishikawa cells during normoglycemia by metformin and particularly insulin. Surprisingly, downregulation was reduced when both substances were combined. On protein level, favorable COL1A1 downregulation was observed with metformin and insulin alone and again particularly after combination in a normoglycemic milieu in Ishikawa cells. During hyperglycemia, a combined treatment also reduced COL1A1 expression, while single treatments did not induce any desired effects. However, elevated glucose concentrations *per se* led to a favorable reduction of COL1A1 levels in untreated cells. In HEC-1A cells, COL1A1 downregulation exclusively occurred after metformin treatment under hyperinsulinemic conditions during normoglycemia, confirming the positive effect of insulin on metformin-mediated COL1A1 regulation.

TIMPs are closely related to MMPs as they act as specific inhibitors on the metastasis-associated proteinases, and therefore, upregulation of TIMP2, TIMP3 and TIMP4 is considered advantageous, while downregulation seems to be favorable for TIMP1 [84]. However, loss of *TIMP1* and associated loss of *PTEN* promoted invasion and migration in prostate cancer *in vivo* [85], while TIMP1 overexpression was associated with cancer progression and poor prognosis in numerous clinical studies [86,87]. TIMP1 levels were also found to be increased in serum and in flushings from women with EC [88,89]. Therefore, in the context of the present study, a *TIMP1* downregulation seems to be more favorable, which was only detected after metformin treatment in Ishikawa cells at normal glucose concentrations and was not affected by additional insulin supplementation. TIMP2, TIMP3 and TIMP4 all inhibit metastasis-related proteinases MMP2 and MMP9, of which MMP9 has already been identified as an interesting target for metformin in the present study. For TIMP2, increased as well as decreased levels were detected in clinical studies [86], but in relevant EC patients, overall survival correlated with enhanced TIMP2 levels [90]. In a study by Dai et al. with HEC-1A cells, inhibition of TIMP2 at mRNA and protein level enhanced MMP2 expression, indicating an unfavorable metastasis-promoting effect [91]. In the present study, *TIMP2* levels were upregulated by metformin in HEC-1A and to a lesser extent in Ishikawa cells only in a hyperglycemic environment. During normoglycemia, *TIMP2* expression was not affected in the HEC-1A cell line and decreased in the premenopausal Ishikawa cell line, indicating a positive influence of glucose on the metformin-induced *TIMP2* regulation. On protein level, TIMP2 was upregulated by 1.0 mmol/L metformin in both cell lines in the presence of elevated glucose concentrations, while metformin induced an unfavorable TIMP2 downregulation during normoglycemia, confirming the results from gene expression analysis and, again, suggesting a positive impact of hyperglycemia. Downregulation of *TIMP3* is associated with cancer progression and poor prognosis and TIMP3 silencing has been found in multiple human cancers [86]. In the context of EC, TIMP3 downregulation was found in high stage endometrioid EC [92] and stimulated growth and invasion in HEC-1B and Ishikawa EC cells *in vitro* [93]. Metformin also led to an increased expression of TIMP1 and TIMP3 in osteoarthritis chondrocytes co-cultured with metformin-treated adipose tissue-derived human mesenchymal stem cells [94]. In the present study with EC cell lines, low-concentration metformin as well as insulin alone induced *TIMP3* downregulation in Ishikawa cells, while 1.0 mmol/L metformin slightly enhanced *TIMP3* expression both under normo-and hyperglycemic conditions; the *TIMP3* gene could not be detected in HEC-1A cells. Like TIMP2, TIMP4 was found to be up-as well as downregulated in tumors depending on the type of cancer [86]. In EC, *TIMP4* transcript levels were lower [95], whereas high TIMP4 protein expression was detected in endometrial tumor tissue in correlation with myometrial invasion, suggesting a key role in endometrial tumor progression [96]. In the present study, 1.0 mmol/L metformin induced *TIMP4* upregulation in HEC-1A and Ishikawa cells in a hyperglycemic environment, suggesting a positive influence of glucose, as seen for *TIMP2* before. Supplementation with insulin further increased *TIMP4* expression only in HEC-1A cells irrespective of changes in glucose concentration.

As a key regulator of the actin cytoskeleton, RAC1 is involved in actin reorganization, which is required for proliferation and migration. *RAC1* downregulation was shown to reduce metastasis [[97], [98], [99]], while *RAC1* activation promoted tumor growth [100]. Metformin reduced RAC1 protein expression and cell migration in prostate cancer cell lines in a study by Dirat et al. [101] as well as in a keratinocyte cell line in a study by Hakimee and colleagues [102]. In the present study, *RAC1* transcript expression has been lowered by 1.0 mmol/L metformin in a hyperglycemic environment in HEC-1A cells, while the same effect was observed in Ishikawa cells only at normal glucose levels, indicating a varying impact of glucose in pre- and postmenopausal settings.

*CDKN1A* acts as a tumor suppressor via G_1_ cell cycle arrest, leading to growth arrest, senescence or apoptosis, but *CDKN1A* inhibits apoptosis and promotes cell proliferation in p53-deficient tumors [[103], [104], [105]]; the HEC-1A as well as the Ishikawa cell lines were found to be *TP53*- as well as p53-positive [[106], [107], [108], [109]]. Additionally, 17–45% of EC tissue samples were p53-positive in various studies [110]. In the present study, metformin upregulated *CDKN1A* transcripts in HEC-1A and Ishikawa cells in the presence and absence of increased insulin levels only under hyperglycemic conditions, while normoglycemia had an unfavorable impact. On protein level, CDKN1A expression was only minimally affected under any test condition in the premenopausal Ishikawa model. In HEC-1A cells, metformin-induced favorable CDKN1A upregulation occurred only during normoglycemia, but not hyperglycemia, which was in contrast to *CDKN1A* transcript regulation. Favorable metformin effects in a normoglycemic environment are particularly interesting for clinical settings, as metformin will normalize glucose levels in patients with hyperglycemia due to its known indirect effects that are induced via blockage of gluconeogenesis. However, high glucose levels *per se* led to an increase in CDKN1A expression in untreated control samples.

CEACAM1 plays a role in adhesion and in pathways related to survival, differentiation as well as growth and it has been suggested to act as a tumor suppressor [111]. *CEACAM1* loss is associated with poor prognosis in gastric cancer patients [112], while overexpression suppressed proliferation, induced cell apoptosis and inhibited migration in multiple myeloma cell lines *in vitro* [111]. On the other hand, CEACAM1 was found to be pro-angiogenic *in vivo* [113] and to stimulate cellular metastasis in various cancer types [114]. In EC and other malignancies, *CEACAM1* expression was downregulated [115,116]. In the present study, favorable *CEACAM1* upregulation was observed after metformin treatment in HEC-1A cells under hyperglycemic conditions, which was even increased when the biguanide was given together with insulin. It has to be noted that *CEACAM1* levels were substantially lower under any tested condition in a hyperglycemic environment when compared with the respective normoglycemic counterpart, indicating a remarkable negative impact of glucose on *CEACAM1* expression. In the Ishikawa premenopausal model, *CEACAM1* expression could not be detected.

IGF1 triggers proliferation and enhances survival upon binding to the IGF1 receptor (IGF1R) [117]. The IGF system plays an important role in estrogen-induced EC [118,119] and high *IGF1* levels stimulate proliferation, migration as well as invasion, and thus promote tumor growth, angiogenesis and metastasis [120]. In HEC-1A cells, *IGF1* expression was reduced by metformin with and without additional insulin in a hyperglycemic milieu in our study, while decreased *IGF1* levels were observed under any tested condition in normoglycemic media in Ishikawa cells, suggesting a varying influence of glucose on metformin-induced effects in a pre-versus a postmenopausal model.

Upon activation during early stage of the extrinsic apoptosis pathway, the initiator caspase CASP8 activates effector caspases 3 and 7. CASP8 is also associated with the intrinsic apoptosis pathway via activation of BH3-interacting domain death agonist (BID) [121]. In tumor cells, CASP8 induces apoptosis and inhibits proliferation [122] and metformin was found to increase CASP8 expression in A498 renal carcinoma cells at 7.5 mmol/L after 24 h [123] as well as in several pancreatic cancer cell lines at 30.0 mmol/L *in vitro* [124]. In the present study, *CASP8* expression was decreased after metformin treatment and in particular after insulin supplementation in normoglycemic media, while no CASP8 regulation was detected in a hyperglycemic milieu, indicating a negative metformin effect on apoptosis induction at normal glucose levels.

BCL2L11 is involved in the intrinsic apoptosis pathway as a pro-apoptotic regulator and overexpression inhibited tumor growth and increased apoptosis induction [125]. In several studies, metformin increased BCL2L11 expression and, for instance, inhibited growth of EC cell line RL95-2 after treatment with 4.0 mmol/L for 48 h [126], induced apoptosis at 1.0 mmol/L after 48 h in H1975 and PC-9 lung cancer cell lines [127] or inhibited proliferation of esophageal cancer cell lines Eca109 and EC9706 at 10.0–20.0 mmol/L after 24 h [128]. In the present study, *BCL2L1*1 has been upregulated after metformin treatment with and without insulin in a hyperglycemic milieu in HEC-1A, but not in Ishikawa cells. The BCL2L11 protein was upregulated by metformin and insulin alone in HEC-1A cells irrespective of the glucose concentration, but a combined treatment induced the desired effect only during normoglycemia. In Ishikawa cells, only treatment with metformin or insulin alone led to favorable BCL2L11 upregulation.

The influence of glucose levels on metformin-induced anti-tumor effects seems to be crucial [129], which has also been shown for some of the abovementioned genes and proteins in the present study. Therefore, our results confirmed the impact of varying concentrations of glucose on the effectiveness of metformin's anti-cancer activity. In clinical settings, metformin-treated diabetic breast cancer patients had a better clinical outcome compared to non-treated patients [130] and the drug significantly improved overall and progression-free survival of patients with T2DM [131]. In the context of EC, there was a lower risk for EC development in women with T2DM when metformin was applied [132]. However, metformin did not lower the risk for tumor development in patients with T2DM in another study, although the drug decreased the risk for diabetic patients to develop other gynecological cancers, particularly of the cervix [133]. Data from other clinical studies have also been inconsistent, likely due to inhomogeneities between patient groups with regard to age, body mass index (BMI), cancer subtypes and cancer stages, as well as preexisting metabolic diseases. Such deviating results were found for overall and progression-free survival, tumor recurrence and lower risk for cancer development in diabetic EC patients after metformin administration [[134], [135], [136]].

In summary, metformin induced favorable regulation of cancer- and metastasis-related genes and proteins in the present study and was able to reduce or cancel negative insulin-induced effects, particularly in a normoglycemic environment, which could be relevant for prediabetic patients with insulin resistance. However, the current research was designed as an *in vitro* study and therefore the predictability of *in vivo* effects is limited. Metformin concentrations applied *in vitro* (0.1 and 1.0 mmol/L) may deviate greatly from clinical doses as well as plasma or tissue levels (typical plasma levels during T2DM therapy are between 1 and 20 μmol/L [137,138]) reached in patients and may even exceed lethal doses, if applied *in vivo*. A possible explanation could be high concentrations of nutrients and growth factors in the culture media, combined with a fluctuating expression of OCT1 (organic cation transporter 1), which is responsible for the cellular uptake of metformin [139]. Also, factors such as cellular uptake, interactions between cell types, tumor microenvironment, or drug stability are different in an *in vivo* setting, but these are general drawbacks of *in vitro* models. Yet, results of the current screening approach were intended to identify potential key targets that contribute to the anti-cancer activity of metformin in pre- and postmenopausal EC under consideration of different environmental influences. Nevertheless, further research will be necessary in order to confirm the results and their potential clinical relevance in *vitro* studies with concentrations in a clinically applicable low micromolar range and in *vivo* studies.

Elevated glucose levels tend to have a positive impact on metformin-mediated effects or prevented negative effects that have been observed under normoglycemia for single molecular targets. However, these effects did not transfer to an overall cellular level, where effects on proliferation, viability, clonogenicity or migration were similar or less favorable under high glucose conditions in a recent study of our group with HEC-1A and Ishikawa EC cell lines [12]. An unfavorable impact of high glucose levels on metformin-induced effects on proliferation, cell cycle arrest or apoptosis induction has also been observed in other *in vitro* studies, e.g. in breast cancer cells [[140], [141], [142]]. Special attention should be given to candidates that were positively regulated by metformin in a normoglycemic environment, as metformin will normalize glucose levels in hyperglycemic and diabetic patients due to its indirect effects induced via blockage of gluconeogenesis.

Metformin-induced changes in gene regulation did not necessarily reflect altered protein expression and are therefore not suitable as general predictive markers. Furthermore, metformin regulated several genes and proteins differentially in pre- and postmenopausal EC models. It also has to be noted that no validation of the functional importance of the identified target genes has been carried out in the present *in vitro* study; knock-down or knock-out of the selected candidate genes would help evaluate the effect of metformin-induced expression changes on cellular functions, e.g. proliferation, migration or viability.

## 5 Conclusions

In the present study, genes and proteins were identified that might be involved in the anti-cancer mechanism of metformin in EC. However, further research will be needed in order to perform pathway analysis, including upstream regulators and downstream targets of the selected candidates. The following conclusions were drawn based on the presented data: Firstly, metformin-induced regulations were glucose-dependent and were interestingly more favorable in a hyperglycemic environment for some molecular targets. Secondly, favorable gene regulations could not be easily extrapolated to protein level and could not reliably predict protein regulation as nothing is known about parameters such as promoter strength, translation efficiency or transcript half-life. Thirdly, metformin reduced or canceled unfavorable regulations that have been induced by elevated insulin levels in a hyperinsulinemic setting in some cases. And finally, in the context of EC, metformin-induced effects varied between pre- and postmenopausal cell lines, indicating a variable sensitivity to the drug due to hormone-induced differences and suggesting deviating outcomes with varying tumor types and tumor stages. With the presented data, we contribute to a better understanding of the anti-cancer activity of metformin as well as its underlying mechanism of action in EC cells. Although further research will be necessary to confirm the data, the influence of different environmental settings on metformin-induced effects could be highlighted with the presented *in vitro* results.

## Author contribution statement

Carsten Lange: Conceived and designed the experiments; Performed the experiments; Analyzed and interpreted the data; Wrote the paper.

Jana Brüggemann; Theresa Thüner: Performed the experiments; Analyzed and interpreted the data; Wrote the paper.

Julia Jauckus: Performed the experiments; Analyzed and interpreted the data.

Thomas Strowitzki: Contributed reagents, materials, analysis tools or data.

Ariane Germeyer; Conceived and designed the experiments; Analyzed and interpreted the data; Contributed reagents, materials, analysis tools or data; Wrote the paper.

## Data availability statement

Data included in article/supp. material/referenced in article.

## Acknowledgments

**Acknowledgments** The authors thank Prof. Alexander Enk and Prof. Karsten Mahnke (Department of Dermatology, University Hospital, Ruprecht-Karls University of Heidelberg, Heidelberg, Germany) for providing the ECL imaging system. For the publication fee we acknowledge financial support by Deutsche Forschungsgemeinschaft within the funding programme "Open Access Publikationskosten" as well as by Heidelberg University.

## Declaration of competing interest

The authors declare that they have no known competing financial interests or personal relationships that could have appeared to influence the work reported in this paper.

## Abbreviations

AKT, PKB: protein kinase B
AMPK: 5′ adenosine monophosphate-activated protein kinase
AMV: avian myeloblastosis virus
APC: adenomatous polyposis coli
BCA: bicinchoninic acid
BCL2L11, BIM: Bcl-2-like protein 11
BID: BH3-interacting domain death agonist
BMI: body mass index
BSA: bovine serum albumin
CASP8: caspase 8
CDH1: cadherin 1, *E*-cadherin
CDKN1A, p21^Waf1/Cip1^: cyclin-dependent kinase inhibitor 1 A
CEACAM1, BGP1, CD66a: carcinoembryonic antigen-related cell adhesion molecule 1
COL1A1: collagen type 1 alpha 1
C_T_ value: cycle threshold value
CTNNA1: catenin alpha-1
DTT: dithiothreitol
E2: β-estradiol
EC: endometrial cancer
ECL: enhanced chemiluminescence
ECM: extracellular matrix
EDTA: ethylenediaminetetraacetic acid
EMT: epithelial-mesenchymal transition
ERα/β: estrogen receptor α/β
FBS: fetal bovine serum
FC: fold-change
GPER: G protein-coupled receptor 1
HRP: horseradish peroxidase
IFG1R: IGF1 receptor
IGF1: insulin-like growth factor 1
MEM: Eagle's minimal essential medium
MMP2/9/10: matrix metalloproteinase 2/9/10, gelatinase A/B/stromelysin 2
MTT: 3-(4,5-dimethylthiazol-2-yl)-2,5-diphenyltetrazolium bromide
NG/HG: normo-/hyperglycemia
OCT1: organic cation transporter 1
PBS: phosphate-buffered saline
PCOS: polycystic ovary syndrome
PI3K: phosphoinositide 3-kinase
PIC: protease inhibitor cocktail
PR: progesterone receptor
PTEN: phosphatase and tensin homolog
PVDF: polyvinylidene difluoride
RAC1: ras-related C3 botulinum toxin substrate 1
Rag GTPase: ras-related guanosine triphosphate hydrolase
T2DM: type 2 diabetes mellitus
TGFB1: transforming growth factor beta 1
TIMP1/2/3/4: tissue inhibitor of metalloproteinases 1/2/3/4
UNG: *N*-glycosylase

## References

[1] Shao R., Li X., Feng Y., Lin J.F., Billig H. (2014). Direct effects of metformin in the endometrium: a hypothetical mechanism for the treatment of women with PCOS and endometrial carcinoma. J. Exp. Clin. Cancer Res..

[2] O'Connor K.A., Ferrell R.J., Brindle E., Shofer J., Holman D.J., Miller R.C. (2009). Total and unopposed estrogen exposure across stages of the transition to menopause. Cancer Epidemiol. Biomarkers Prev..

[3] Modesitt S.C., Geffel D.L., Via J. (2012). Morbidly obese women with and without endometrial cancer: are there differences in measured physical fitness, body composition, or hormones?. Gynecol. Oncol..

[4] Joung K.H., Jeong J.W., Ku B.J. (2015). The association between type 2 diabetes mellitus and women cancer: the epidemiological evidences and putative mechanisms. BioMed Res. Int..

[5] Flory J., Lipska K. (2019). Metformin in 2019. JAMA.

[6] Meireles C.G., Pereira S.A., Valadares L.P., Rego D.F., Simeoni L.A., Guerra E.N.S., Lofrano-Porto A. (2017). Effects of metformin on endometrial cancer: systematic review and meta-analysis. Gynecol. Oncol..

[7] Libby G., Donnelly L.A., Donnan P.T., Alessi D.R., Morris A.D., Evans J.M. (2009). New users of metformin are at low risk of incident cancer: a cohort study among people with type 2 diabetes. Diabetes Care.

[8] Evans J.M.M., Donnelly L.A., Emslie-Smith A.M., Alessi D.R., Morris A.D. (2005). Metformin and reduced risk of cancer in diabetic patients. BMJ.

[9] Ko E.M., Walter P., Jackson A., Clark L., Franasiak J., Bolac C. (2014). Metformin is associated with improved survival in endometrial cancer. Gynecol. Oncol..

[10] Nevadunsky N.S., Van Arsdale A., Strickler H.D., Moadel A., Kaur G., Frimer M. (2014). Metformin use and endometrial cancer survival. Gynecol. Oncol..

[11] Currie C.J., Poole C.D., Jenkins-Jones S., Gale E.A., Johnson J.A., Morgan C.L. (2012). Mortality after incident cancer in people with and without type 2 diabetes: impact of metformin on survival. Diabetes Care.

[12] Machado Weber A., Lange C., Jauckus J., Strowitzki T., Germeyer A. (2021). Long-term metformin effect on endometrial cancer development depending on glucose environment *in vitro*. Open J. Obstet. Gynecol..

[13] Foretz M., Guigas B., Viollet B. (2019). Understanding the glucoregulatory mechanisms of metformin in type 2 diabetes mellitus. Nat. Rev. Endocrinol..

[14] Pernicova I., Korbonits M. (2014). Metformin - mode of action and clinical implications for diabetes and cancer. Nat. Rev. Endocrinol..

[15] Harada N. (2020). Effects of metformin on blood glucose levels and bodyweight mediated through intestinal effects. J Diabetes Investig.

[16] Krauzova E., Tuma P., de Glisezinski I., Stich V., Siklova M. (2018). Metformin does not inhibit exercise-induced lipolysis in adipose tissue in young healthy lean men. Front. Physiol..

[17] Pierotti M.A., Berrino F., Gariboldi M., Melani C., Mogavero A., Negri T. (2013). Targeting metabolism for cancer treatment and prevention: metformin, an old drug with multi-faceted effects. Oncogene.

[18] Kalender A., Selvaraj A., Kim S.Y., Gulati P., Brule S., Viollet B. (2010). Metformin, independent of AMPK, inhibits mTORC1 in a rag GTPase-dependent manner. Cell Metabol..

[19] Kourelis T.V., Siegel R.D. (2012). Metformin and cancer: new applications for an old drug. Med. Oncol..

[20] Vial G., Detaille D., Guigas B. (2019). Role of mitochondria in the mechanism(s) of action of metformin. Front. Endocrinol..

[21] Kuramoto H., Tamura S., Notake Y. (1972). Establishment of a cell line of human endometrial adenocarcinoma *in vitro*. Am. J. Obstet. Gynecol..

[22] Kuramoto H., Hamano M., Imai M. (2002). HEC-1 cells. Hum. Cell.

[23] Hevir-Kene N., Rizner T.L. (2015). The endometrial cancer cell lines Ishikawa and HEC-1A, and the control cell line HIEEC, differ in expression of estrogen biosynthetic and metabolic genes, and in androstenedione and estrone-sulfate metabolism. Chem. Biol. Interact..

[24] Nishida M., Kasahara K., Kaneko M., Iwasaki H., Hayashi K. (1985). Establishment of a new human endometrial adenocarcinoma cell line, Ishikawa cells, containing estrogen and progesterone receptors. Nippon. Sanka Fujinka Gakkai Zasshi.

[25] Nishida M. (2002). The Ishikawa cells from birth to the present. Hum. Cell.

[26] Lu J., Zhang X., Zhang R., Ge Q. (2015). microRNA heterogeneity in endometrial cancer cell lines revealed by deep sequencing. Oncol. Lett..

[27] Czech M.P. (2017). Insulin action and resistance in obesity and type 2 diabetes. Nat. Med..

[28] Yang X., Wang J. (2019). The role of metabolic syndrome in endometrial cancer: a review. Front. Oncol..

[29] Clarke C.L., Sutherland R.L. (1990). Progestin regulation of cellular proliferation. Endocr. Rev..

[30] Liu R., Guan S., Gao Z., Wang J., Xu J., Hao Z. (2021). Pathological hyperinsulinemia and hyperglycemia in the impaired glucose tolerance stage mediate endothelial dysfunction through miR-21, PTEN/AKT/eNOS, and MARK/ET-1 pathways. Front. Endocrinol..

[31] Turner M.C., Martin N.R.W., Player D.J., Ferguson R.A., Wheeler P., Green C.J. (2020). Characterising hyperinsulinemia-induced insulin resistance in human skeletal muscle cells. J. Mol. Endocrinol..

[32] Rossi A., Eid M., Dodgson J., Davies G., Musial B., Wabitsch M. (2020). *In vitro* characterization of the effects of chronic insulin stimulation in mouse 3T3-L1 and human SGBS adipocytes. Adipocyte.

[33] Watanabe N., Kobayashi M., Maegawa H., Ishibashi O., Takata Y., Shigeta Y. (1986). Long-term *in vitro* effects of insulin on insulin binding and glucose transport. Diabetes Res. Clin. Pract..

[34] Lange C., Machado Weber A., Schmidt R., Schroeder C., Strowitzki T., Germeyer A. (2021). Changes in protein expression due to metformin treatment and hyperinsulinemia in a human endometrial cancer cell line. PLoS One.

[35] Machado Weber A., Strowitzki T., Germeyer A. (2018). High glucose levels interferes the endometrial cancer cell response to metformin treatment over time. Br. J. Res..

[36] de Barros Machado A., Dos Reis V., Weber S., Jauckus J., Brum I.S. (2016). von Eye Corleta, H. et al. Proliferation and metastatic potential of endometrial cancer cells in response to metformin treatment in a high versus normal glucose environment. Oncol. Lett..

[37] Laemmli U.K. (1970). Cleavage of structural proteins during the assembly of the head of bacteriophage T4. Nature.

[38] Abramoff M.D., Magalhaes P.J., Ram S.J. (2004). Image processing with ImageJ. Biophot. Int..

[39] Schneider C.A., Rasband W.S., Eliceiri K.W. (2012). NIH Image to ImageJ: 25 years of image analysis. Nat. Methods.

[40] Weigelt B., Warne P.H., Lambros M.B., Reis-Filho J.S., Downward J. (2013). PI3K pathway dependencies in endometrioid endometrial cancer cell lines. Clin. Cancer Res..

[41] Bian X., Gao J., Luo F., Rui C., Zheng T., Wang D. (2018). PTEN deficiency sensitizes endometrioid endometrial cancer to compound PARP-PI3K inhibition but not PARP inhibition as monotherapy. Oncogene.

[42] Kariola R., Abdel-Rahman W.M., Ollikainen M., Butzow R., Peltomaki P., Nystrom M. (2005). APC and beta-catenin protein expression patterns in HNPCC-related endometrial and colorectal cancers. Fam. Cancer.

[43] Pijnenborg J.M., Kisters N., van Engeland M., Dunselman G.A., de Haan J., de Goeij A.F., Groothuis P.G. (2004). APC, beta-catenin, and E-cadherin and the development of recurrent endometrial carcinoma. Int. J. Gynecol. Cancer.

[44] Choi S.H., Estaras C., Moresco J.J., Yates J.R., Jones K.A. (2013). α-Catenin interacts with APC to regulate β-catenin proteolysis and transcriptional repression of Wnt target genes. Genes Dev..

[45] Chi Q., Xu H., Song D., Wang Z., Wang Z., Ma G. (2020). α-E-catenin (CTNNA1) inhibits cell proliferation, invasion and EMT of bladder cancer. Cancer Manag. Res..

[46] Fujimoto J., Ichigo S., Hori M., Tamaya T. (1998). Expressions of E-cadherin and alpha- and beta-catenin mRNAs in uterine endometrial cancers. Eur. J. Gynaecol. Oncol..

[47] Fujimoto J., Ichigo S., Hirose R., Sakaguchi H., Tamaya T. (1997). Suppression of E-cadherin and alpha- and beta-catenin mRNA expression in the metastatic lesions of gynecological cancers. Eur. J. Gynaecol. Oncol..

[48] Mendonsa A.M., Na T.Y., Gumbiner B.M. (2018). E-cadherin in contact inhibition and cancer. Oncogene.

[49] Canel M., Serrels A., Frame M.C., Brunton V.G. (2013). E-cadherin-integrin crosstalk in cancer invasion and metastasis. J. Cell Sci..

[50] Florescu M.M., Pirici D., Simionescu C.E., Stepan A.E., Margaritescu C., Tudorache Ş., Ciurea R.N. (2016). E-cadherin and β-catenin immunoexpression in endometrioid endometrial carcinoma. Rom. J. Morphol. Embryol..

[51] Laskov I., Abou-Nader P., Amin O., Philip C.A., Beauchamp M.C., Yasmeen A., Gotlieb W.H. (2016). Metformin increases E-cadherin in tumors of diabetic patients with endometrial cancer and suppresses epithelial-mesenchymal transition in endometrial cancer cell lines. Int. J. Gynecol. Cancer.

[52] Qiang P., Shao Y., Sun Y.P., Zhang J., Chen L.J. (2019). Metformin inhibits proliferation and migration of endometrial cancer cells through regulating PI3K/AKT/MDM2 pathway. Eur. Rev. Med. Pharmacol. Sci..

[53] Vogelmann R., Nguyen-Tat M.D., Giehl K., Adler G., Wedlich D., Menke A. (2005). TGFbeta-induced downregulation of E-cadherin-based cell-cell adhesion depends on PI3-kinase and PTEN. J. Cell Sci..

[54] Ha B., Lee E.B., Cui J., Kim Y., Jang H.H. (2015). YB-1 overexpression promotes a TGF-β1-induced epithelial-mesenchymal transition via Akt activation. Biochem. Biophys. Res. Commun..

[55] Ingman W.V., Robertson S.A. (2009). The essential roles of TGFB1 in reproduction. Cytokine Growth Factor Rev..

[56] Xie R., Schlumbrecht M.P., Shipley G.L., Xie S., Bassett R.L., Broaddus R.R. (2009). S100A4 mediates endometrial cancer invasion and is a target of TGF-beta1 signaling. Lab. Invest..

[57] Xiong S., Klausen C., Cheng J.C., Leung P.C.K. (2017). TGFβ1 induces endometrial cancer cell adhesion and migration by up-regulating integrin αvβ3 via SMAD-independent MEK-ERK1/2 signaling. Cell. Signal..

[58] Croxtall J.D., Jamil A., Ayub M., Colletta A.A., White J.O. (1992). TGF-beta stimulation of endometrial and breast-cancer cell growth. Int. J. Cancer.

[59] Xiao H., Zhang J., Xu Z., Feng Y., Zhang M., Liu J. (2016). Metformin is a novel suppressor for transforming growth factor (TGF)-β1. Sci. Rep..

[60] Heydarpour F., Sajadimajd S., Mirzarazi E., Haratipour P., Joshi T., Farzaei M.H. (2020). Involvement of TGF-β and autophagy pathways in pathogenesis of diabetes: a comprehensive review on biological and pharmacological insights. Front. Pharmacol..

[61] Persad A., Venkateswaran G., Hao L., Garcia M.E., Yoon J., Sidhu J., Persad S. (2016). Active β-catenin is regulated by the PTEN/PI3 kinase pathway: a role for protein phosphatase PP2A. Genes Cancer.

[62] Fournier M.V., Fata J.E., Martin K.J., Yaswen P., Bissell M.J. (2009). Interaction of E-cadherin and PTEN regulates morphogenesis and growth arrest in human mammary epithelial cells. Cancer Res..

[63] Yokoyama Y., Wan X., Shinohara A., Takahashi S., Takahashi Y., Niwa K., Tamaya T. (2000). Expression of PTEN and PTEN pseudogene in endometrial carcinoma. Int. J. Mol. Med..

[64] Xue P., Fan W., Diao Z., Li Y., Kong C., Dai X. (2020). Up-regulation of PTEN via LPS/AP-1/NF-κB pathway inhibits trophoblast invasion contributing to preeclampsia. Mol. Immunol..

[65] Zhang L., Kong L., Yang Y. (2020). miR-18a inhibitor suppresses leukemia cell proliferation by upregulation of PTEN expression. Med. Sci. Mon. Int. Med. J. Exp. Clin. Res..

[66] Pabona J.M.P., Burnett A.F., Brown D.M., Quick C.M., Simmen F.A., Montales M.T.E. (2020). Metformin promotes anti-tumor biomarkers in human endometrial cancer cells. Reprod. Sci..

[67] Lee S.K., Lee J.O., Kim J.H., Kim S.J., You G.Y., Moon J.W. (2011). Metformin sensitizes insulin signaling through AMPK-mediated pten down-regulation in preadipocyte 3T3-L1 cells. J. Cell. Biochem..

[68] Del Campo J.A., Garcia-Valdecasas M., Gil-Gomez A., Rojas Á., Gallego P., Ampuero J. (2018). Simvastatin and metformin inhibit cell growth in hepatitis C virus infected cells via mTOR increasing PTEN and autophagy. PLoS One.

[69] Gao X., Qiao X., Xing X., Huang J., Qian J., Wang Y. (2020). Matrix stiffness-upregulated microRNA-17-5p attenuates the intervention effects of metformin on HCC invasion and metastasis by targeting the PTEN/PI3K/Akt pathway. Front. Oncol..

[70] Park M.-J., Kim M.-S., Park I.-C., Kang H.-S., Yoo H., Park S.H. (2002). PTEN suppresses hyaluronic acid-induced matrix metalloproteinase-9 expression in U87MG glioblastoma cells through focal adhesion kinase dephosphorylation. Cancer Res..

[71] Moon S.K., Kim H.M., Kim C.H. (2004). PTEN induces G1 cell cycle arrest and inhibits MMP-9 expression via the regulation of NF-kappaB and AP-1 in vascular smooth muscle cells. Arch. Biochem. Biophys..

[72] Moroz A., Delella F.K., Almeida R., Lacorte L.M., Favaro W.J., Deffune E., Felisbino S.L. (2013). Finasteride inhibits human prostate cancer cell invasion through MMP2 and MMP9 downregulation. PLoS One.

[73] Li H., Qiu Z., Li F., Wang C. (2017). The relationship between MMP-2 and MMP-9 expression levels with breast cancer incidence and prognosis. Oncol. Lett..

[74] Liu C., Li Y., Hu S., Chen Y., Gao L., Liu D. (2018). Clinical significance of matrix metalloproteinase-2 in endometrial cancer: a systematic review and meta-analysis. Medicine.

[75] Nothnick W.B. (2008). Regulation of uterine matrix metalloproteinase-9 and the role of microRNAs. Semin. Reprod. Med..

[76] Tamakoshi K., Kikkawa F., Nawa A., Ishikawa H., Mizuno K., Tamakoshi A. (1995). Characterization of extracellular matrix-degrading proteinase and its inhibitor in gynecologic cancer tissues with clinically different metastatic form. Cancer.

[77] Jang S.Y., Kim A., Kim J.K., Kim C., Cho Y.H., Kim J.H. (2014). Metformin inhibits tumor cell migration via down-regulation of MMP9 in tamoxifen-resistant breast cancer cells. Anticancer Res..

[78] Shi X., Chen Z., Hu X., Luo M., Sun Z., Li J. (2016). AJUBA promotes the migration and invasion of esophageal squamous cell carcinoma cells through upregulation of MMP10 and MMP13 expression. Oncotarget.

[79] Klupp F., Neumann L., Kahlert C., Diers J., Halama N., Franz C. (2016). Serum MMP7, MMP10 and MMP12 level as negative prognostic markers in colon cancer patients. BMC Cancer.

[80] Wang Q., Yu J. (2018). miR-129-5p suppresses gastric cancer cell invasion and proliferation by inhibiting COL1A1. Biochem. Cell. Biol..

[81] Liu J., Shen J.X., Wu H.T., Li X.L., Wen X.F., Du C.W., Zhang G.J. (2018). Collagen 1A1 (COL1A1) promotes metastasis of breast cancer and is a potential therapeutic target. Discov. Med..

[82] Ma H.P., Chang H.L., Bamodu O.A., Yadav V.K., Huang T.Y., Wu A.T.H. (2019). Collagen 1A1 (COL1A1) is a reliable biomarker and putative therapeutic target for hepatocellular carcinogenesis and metastasis. Cancers.

[83] Zhu Y., Shi L., Chen P., Zhang Y., Zhu T. (2020). Identification of six candidate genes for endometrial carcinoma by bioinformatics analysis. World J. Surg. Oncol..

[84] Cabral-Pacheco G.A., Garza-Veloz I., Castruita-De la Rosa C., Ramirez-Acuna J.M., Perez-Romero B.A., Guerrero-Rodriguez J.F. (2020). The roles of matrix metalloproteinases and their inhibitors in human diseases. Int. J. Mol. Sci..

[85] Guccini I., Revandkar A., D'Ambrosio M., Colucci M., Pasquini E., Mosole S. (2021). Senescence reprogramming by TIMP1 deficiency promotes prostate cancer metastasis. Cancer Cell.

[86] Jackson H.W., Defamie V., Waterhouse P., Khokha R. (2017). TIMPs: versatile extracellular regulators in cancer. Nat. Rev. Cancer.

[87] Song G., Xu S., Zhang H., Wang Y., Xiao C., Jiang T. (2016). TIMP1 is a prognostic marker for the progression and metastasis of colon cancer through FAK-PI3K/AKT and MAPK pathway. J. Exp. Clin. Cancer Res..

[88] Laird S.M., Dalton C.F., Okon M.A., Bunning R.A., Marshall R., Li T.C. (1999). Metalloproteinases and tissue inhibitor of metalloproteinase 1 (TIMP-1) in endometrial flushings from pre- and post-menopausal women and from women with endometrial adenocarcinoma. J. Reprod. Fertil..

[89] Honkavuori M., Talvensaari-Mattila A., Puistola U., Turpeenniemi-Hujanen T., Santala M. (2008). High serum TIMP-1 is associated with adverse prognosis in endometrial carcinoma. Anticancer Res..

[90] Honkavuori-Toivola M., Talvensaari-Mattila A., Soini Y., Turpeenniemi-Hujanen T., Santala M. (2012). Immunoreactivity for TIMP-2 is associated with a favorable prognosis in endometrial carcinoma. Tumour Biol.

[91] Dai Y., Xia W., Song T., Su X., Li J., Li S. (2013). microRNA-200b is overexpressed in endometrial adenocarcinomas and enhances MMP2 activity by downregulating TIMP2 in human endometrial cancer cell line HEC-1A cells. Nucleic Acid Therapeut..

[92] Catasus L., Pons C., Munoz J., Espinosa I., Prat J. (2013). Promoter hypermethylation contributes to TIMP3 down-regulation in high stage endometrioid endometrial carcinomas. Histopathology.

[93] Yu D., Zhou H., Xun Q., Xu X., Ling J., Hu Y. (2012). microRNA-103 regulates the growth and invasion of endometrial cancer cells through the downregulation of tissue inhibitor of metalloproteinase 3. Oncol. Lett..

[94] Park M.J., Moon S.J., Baek J.A., Lee E.J., Jung K.A., Kim E.K. (2019). Metformin augments anti-inflammatory and chondroprotective properties of mesenchymal stem cells in experimental osteoarthritis. J. Immunol..

[95] Pilka R., Domanski H., Hansson S., Eriksson P., Casslen B. (2004). Endometrial TIMP-4 mRNA is high at midcycle and in hyperplasia, but down-regulated in malignant tumours. Coordinated expression with MMP-26. Mol. Hum. Reprod..

[96] Tunuguntla R., Ripley D., Sang Q.X., Chegini N. (2003). Expression of matrix metalloproteinase-26 and tissue inhibitors of metalloproteinases TIMP-3 and -4 in benign endometrium and endometrial cancer. Gynecol. Oncol..

[97] Alcalde J., Munk M., Gonzalez-Munoz M., Panina S., Berchtold M.W., Villalobo A. (2021). Calmodulin downregulation in conditional knockout HeLa cells inhibits cell migration. Arch. Biochem. Biophys..

[98] Lashgarian H.E., Adamii V., Ghorbanzadeh V., Chodari L., Kamali F., Akbari S., Dariushnejad H. (2020). Silibinin inhibit cell migration through downregulation of RAC1 gene expression in highly metastatic breast cancer cell line. Drug Res..

[99] Salker M.S., Schierbaum N., Alowayed N., Singh Y., Mack A.F., Stournaras C. (2016). LeftyA decreases actin polymerization and stiffness in human endometrial cancer cells. Sci. Rep..

[100] Zhu Z., Yu Z., Rong Z., Luo Z., Zhang J., Qiu Z., Huang C. (2019). The novel GINS4 axis promotes gastric cancer growth and progression by activating Rac1 and CDC42. Theranostics.

[101] Dirat B., Ader I., Golzio M., Massa F., Mettouchi A., Laurent K. (2015). Inhibition of the GTPase Rac1 mediates the antimigratory effects of metformin in prostate cancer cells. Mol. Cancer Therapeut..

[102] Hakimee H., Hutamekalin P., Tanasawet S., Chonpathompikunlert P., Tipmanee V., Sukketsiri W. (2019). Metformin inhibit cervical cancer migration by suppressing the FAK/Akt signaling pathway. Asian Pac. J. Cancer Prev. APJCP.

[103] Georgakilas A.G., Martin O.A., Bonner W.M. (2017). p21: a two-faced genome guardian. Trends Mol. Med..

[104] Parveen A., Akash M.S., Rehman K., Kyunn W.W. (2016). Dual role of p21 in the progression of cancer and its treatment. Crit. Rev. Eukaryot. Gene Expr..

[105] Gartel A.L. (2009). p21^WAF1/CIP1^ and cancer: a shifting paradigm?. Biofactors.

[106] Yaginuma Y., Westphal H. (1991). Analysis of the p53 gene in human uterine carcinoma cell lines. Cancer Res..

[107] Ma Y., Jiang J., Zhang Y., Ding Y., Xu T., Lu B. (2017). IGFBP-rP1 acts as a potential tumor suppressor via the suppression of ERK signaling pathway in endometrial cancer cells. Mol. Med. Rep..

[108] Chen H.Y., Cheng W.P., Chiang Y.F., Hong Y.H., Ali M., Huang T.C. (2021). Hinokitiol exhibits antitumor properties through induction of ROS-mediated apoptosis and p53-driven cell-cycle arrest in endometrial cancer cell lines (Ishikawa, HEC-1A, KLE). Int. J. Mol. Sci..

[109] Zhang X., Cheng L., Minn K., Madan R., Godwin A.K., Shridhar V., Chien J. (2014). Targeting of mutant p53-induced FoxM1 with thiostrepton induces cytotoxicity and enhances carboplatin sensitivity in cancer cells. Oncotarget.

[110] Nakamura M., Obata T., Daikoku T., Fujiwara H. (2019). The association and significance of p53 in gynecologic cancers: the potential of targeted therapy. Int. J. Mol. Sci..

[111] Xu J., Liu B., Ma S., Zhang J., Ji Y., Xu L. (2018). Characterizing the tumor suppressor role of CEACAM1 in multiple myeloma. Cell. Physiol. Biochem..

[112] Takeuchi A., Yokoyama S., Nakamori M., Nakamura M., Ojima T., Yamaguchi S. (2019). Loss of CEACAM1 is associated with poor prognosis and peritoneal dissemination of patients with gastric cancer. Sci. Rep..

[113] Gerstel D., Wegwitz F., Jannasch K., Ludewig P., Scheike K., Alves F. (2011). CEACAM1 creates a pro-angiogenic tumor microenvironment that supports tumor vessel maturation. Oncogene.

[114] Ling Y., Wang J., Wang L., Hou J., Qian P., Xiang-dong W. (2015). Roles of CEACAM1 in cell communication and signaling of lung cancer and other diseases. Cancer Metastasis Rev..

[115] Bamberger A.M., Briese J., Gotze J., Erdmann I., Schulte H.M., Wagener C., Nollau P. (2006). Stimulation of CEACAM1 expression by 12-O-tetradecanoylphorbol-13-acetate (TPA) and calcium ionophore A23187 in endometrial carcinoma cells. Carcinogenesis.

[116] Bamberger A.M., Riethdorf L., Nollau P., Naumann M., Erdmann I., Gotze J. (1998). Dysregulated expression of CD66a (BGP, C-CAM), an adhesion molecule of the CEA family, in endometrial cancer. Am. J. Pathol..

[117] Weroha S.J., Haluska P. (2012). The insulin-like growth factor system in cancer. Endocrinol Metab. Clin. N. Am..

[118] Liang Y.-J., Hao Q., Zhang H.-M., Wu Y.-Z., Wang J.-D. (2012). Insulin-like growth factors in endometrioid adenocarcinoma: correlation with clinico-pathological features and estrogen receptor expression. BMC Cancer.

[119] Bruchim I., Sarfstein R., Werner H. (2014). The IGF hormonal network in endometrial cancer: functions, regulation, and targeting approaches. Front. Endocrinol..

[120] Hua H., Kong Q., Yin J., Zhang J., Jiang Y. (2020). Insulin-like growth factor receptor signaling in tumorigenesis and drug resistance: a challenge for cancer therapy. J. Hematol. Oncol..

[121] Kantari C., Walczak H. (2011). Caspase-8 and Bid: caught in the act between death receptors and mitochondria. Biochim. Biophys. Acta.

[122] Wang H.B., Li T., Ma D.Z., Ji Y.X., Zhi H. (2017). Overexpression of FADD and Caspase-8 inhibits proliferation and promotes apoptosis of human glioblastoma cells. Biomed. Pharmacother..

[123] Jang J.-H., Song I.-H., Sung E.-G., Lee T.-J., Kim J.-Y. (2018). Metformin-induced apoptosis facilitates degradation of the cellular caspase 8 (FLICE)-like inhibitory protein through a caspase-dependent pathway in human renal cell carcinoma A498 cells. Oncol. Lett..

[124] Wang L.W., Li Z.S., Zou D.W., Jin Z.D., Gao J., Xu G.M. (2008). Metformin induces apoptosis of pancreatic cancer cells. World J. Gastroenterol..

[125] Sur S., Steele R., Shi X., Ray R.B. (2019). miRNA-29b inhibits prostate tumor growth and induces apoptosis by increasing Bim expression. Cells.

[126] Kim J.S., Turbov J., Rosales R., Thaete L.G., Rodriguez G.C. (2019). Combination simvastatin and metformin synergistically inhibits endometrial cancer cell growth. Gynecol. Oncol..

[127] Chen H., Wang Y., Lin C., Lu C., Han R., Jiao L. (2017). Vorinostat and metformin sensitize EGFR-TKI resistant NSCLC cells via BIM-dependent apoptosis induction. Oncotarget.

[128] Tang J.C., An R., Jiang Y.Q., Yang J. (2017). Effects and mechanisms of metformin on the proliferation of esophageal cancer cells *in vitro* and *in vivo*. Cancer Res Treat.

[129] Aljofan M., Riethmacher D. (2019). Anticancer activity of metformin: a systematic review of the literature. Future Sci OA.

[130] Hou G., Zhang S., Zhang X., Wang P., Hao X., Zhang J. (2013). Clinical pathological characteristics and prognostic analysis of 1,013 breast cancer patients with diabetes. Breast Cancer Res. Treat..

[131] Gong H., Chen Y., Zhou D. (2020). Prognostic significance of metformin treatment in endometrial cancer: a meta-analysis. Pharmazie.

[132] Tseng C.-H. (2015). Metformin and endometrial cancer risk in Chinese women with type 2 diabetes mellitus in Taiwan. Gynecol. Oncol..

[133] Wen Q., Zhao Z., Wen J., Zhou J., Wu J., Lei S., Miao Y. (2019). The association between metformin therapy and risk of gynecological cancer in patients: two meta-analyses. Eur. J. Obstet. Gynecol. Reprod. Biol..

[134] Chu D., Wu J., Wang K., Zhao M., Wang C., Li L., Guo R. (2018). Effect of metformin use on the risk and prognosis of endometrial cancer: a systematic review and meta-analysis. BMC Cancer.

[135] Urpilainen E., Arima R., Karihtala P., Puistola U., Ahtikoski A. (2021). Metformin associates with aggressive features of endometrial cancer in women with type 2 diabetes. Anticancer Res..

[136] Al Hilli M.M., Bakkum-Gamez J.N., Mariani A., Cliby W.A., Mc Gree M.E., Weaver A.L. (2016). The effect of diabetes and metformin on clinical outcomes is negligible in risk-adjusted endometrial cancer cohorts. Gynecol. Oncol..

[137] Christensen M.M.H., Hojlund K., Hother-Nielsen O., Stage T.B., Damkier P., Beck-Nielsen H., Brosen K. (2015). Steady-state pharmacokinetics of metformin is independent of the OCT1 genotype in healthy volunteers. Eur. J. Clin. Pharmacol..

[138] Sutkowska E., Fortuna P., Wisniewski J., Sutkowska K., Hodurek P., Gamian A., Kaluza B. (2021). Low metformin dose and its therapeutic serum concentration in prediabetes. Sci. Rep..

[139] Dowling R.J., Niraula S., Stambolic V., Goodwin P.J. (2012). Metformin in cancer: translational challenges. J. Mol. Endocrinol..

[140] Varghese S., Samuel S.M., Varghese E., Kubatka P., Busselberg D. (2019). High glucose represses the anti-proliferative and pro-apoptotic effect of metformin in triple negative Breast cancer cells. Biomolecules.

[141] Menendez J.A., Oliveras-Ferraros C., Cufí S., Corominas-Faja B., Joven J., Martin-Castillo B., Vazquez-Martin A. (2012). Metformin is synthetically lethal with glucose withdrawal in cancer cells. Cell Cycle.

[142] Varghese E., Samuel S.M., Liskova A., Samec M., Kubatka P., Busselberg D. (2020). Targeting glucose metabolism to overcome resistance to anticancer chemotherapy in breast cancer. Cancers.

