# Supplementary material for: Changes in the expression of cancer- and metastasis-related genes and proteins after metformin treatment under different metabolic conditions in endometrial cancer cells
Source: Heliyon. 2023 May 25;9(6):e16678. doi: 10.1016/j.heliyon.2023.e16678 (PMC10258389; doi:10.1016/j.heliyon.2023.e16678)
Supplement: Multimedia component 2 [file mmc2.pdf]

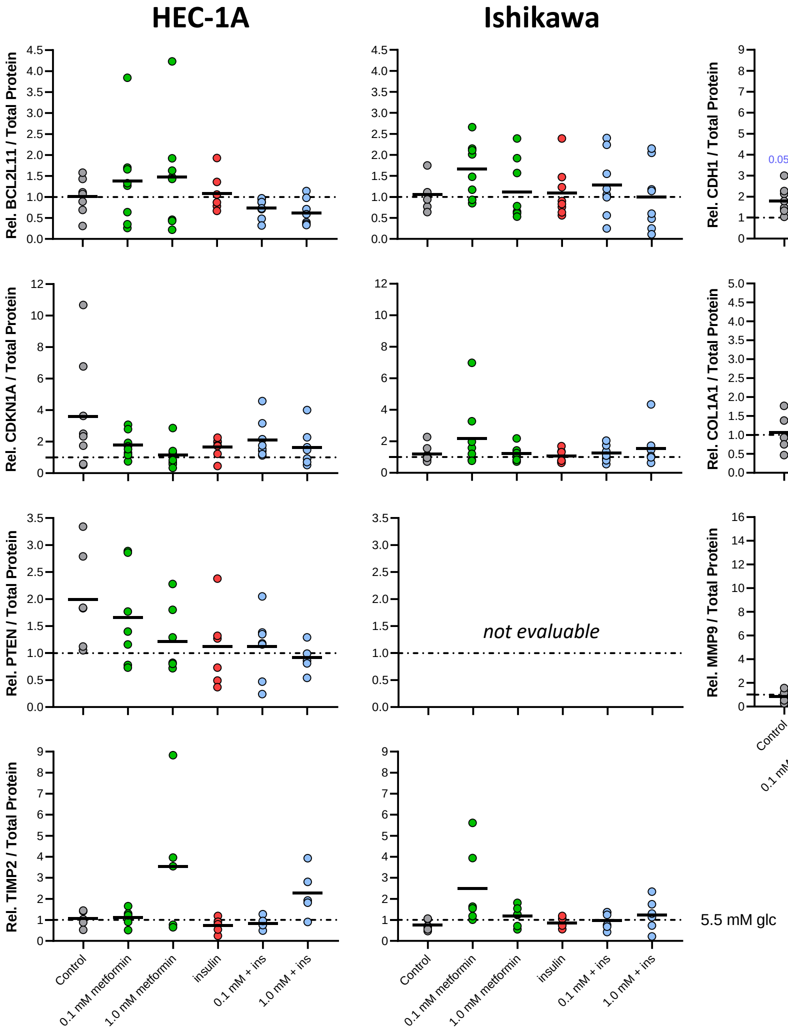

Figure S2. Changes in the expression of selected proteins after treatment of HEC-1A and Ishikawa cells with metformin (green), insulin (red), or a combination of both substances (blue) under normo- or hyperglycemic conditions for 7 d, as detected by subsequent western blot analysis. Semi-quantitative, densitometric analysis was carried out in order to determine the relative normalized protein expression (fold-change). Expression levels under hyperglycemic conditions were calculated relative to the expression in identically treated cells under normoglycemic conditions (fold-changes were set to 1.0 for the normoglycemic samples as indicated by a dotted line). Data presented as dot plots with arithmetic means of at least three independent experiments. Significant differences were determined with a mixed effects model analysis and subsequent Šídák's (analysis of glucose effects between identical treatments) multiple comparison *post-hoc* test; # $p \leq 0.05$ (glucose effect, blue); $p < 0.15$ additionally displayed as values.
