# Supplementary material for: Changes in the expression of cancer- and metastasis-related genes and proteins after metformin treatment under different metabolic conditions in endometrial cancer cells
Source: Heliyon. 2023 May 25;9(6):e16678. doi: 10.1016/j.heliyon.2023.e16678 (PMC10258389; doi:10.1016/j.heliyon.2023.e16678)

Uncropped and unadjusted representative images used for Figure 3

Chemiluminescence

Marker Overlay

HEC-1A

BCL2L11:

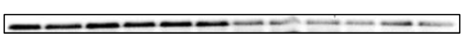

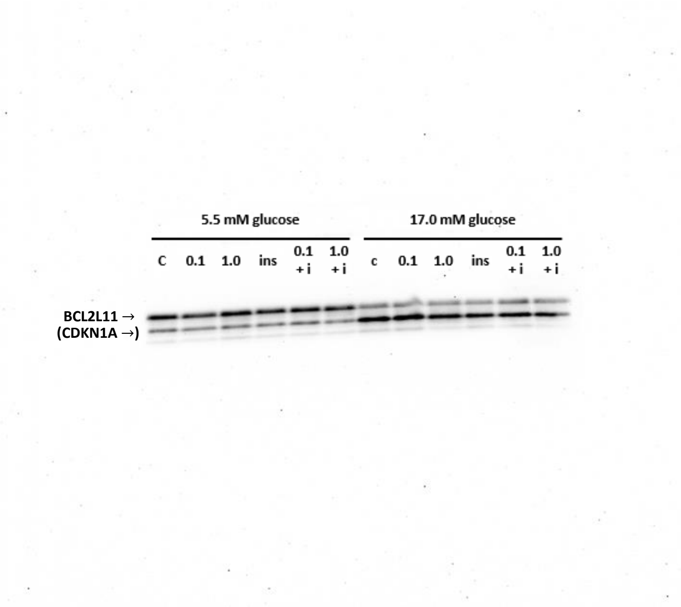

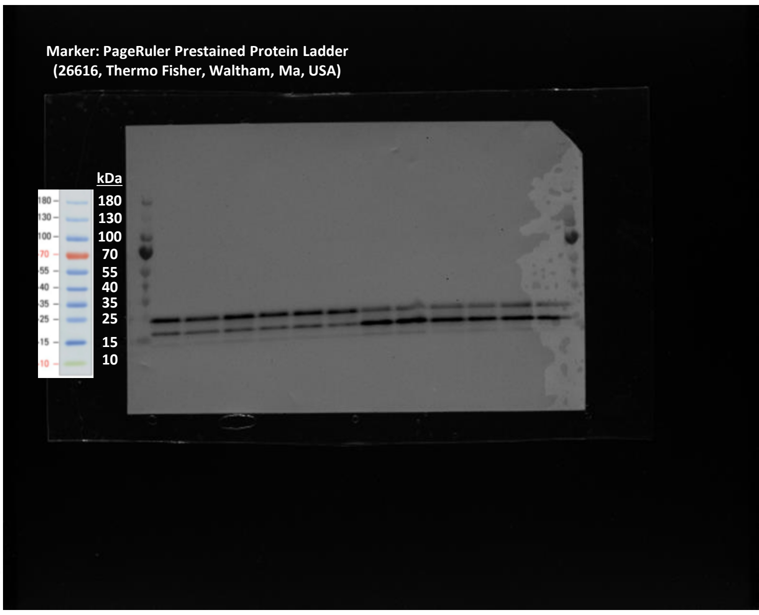

HEC-1A

CDH1:

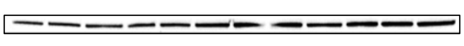

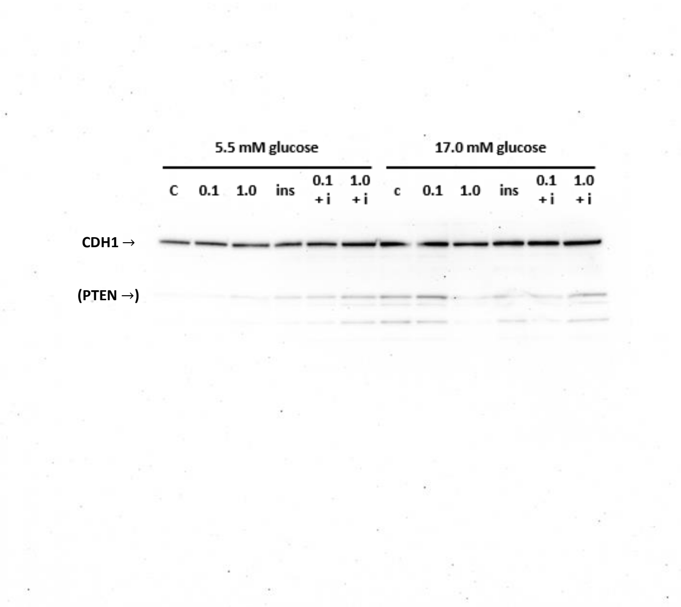

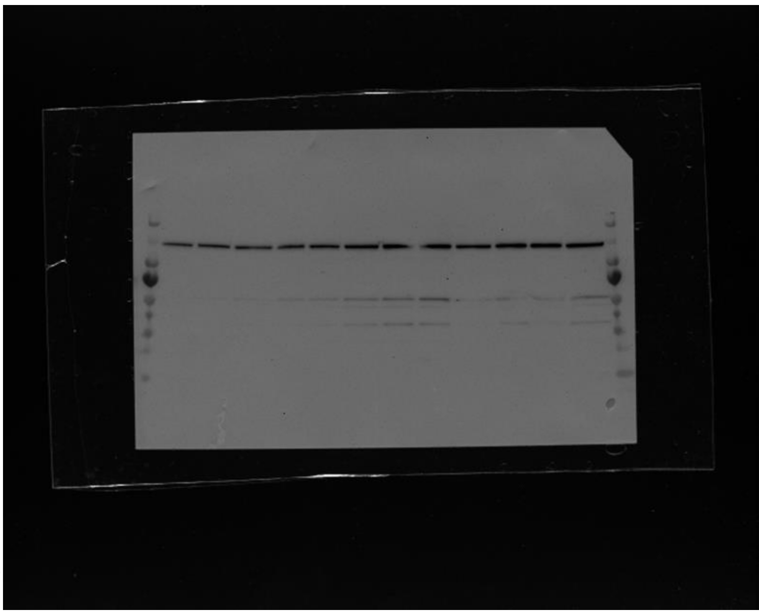

HEC-1A

CDKN1A:

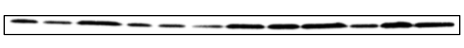

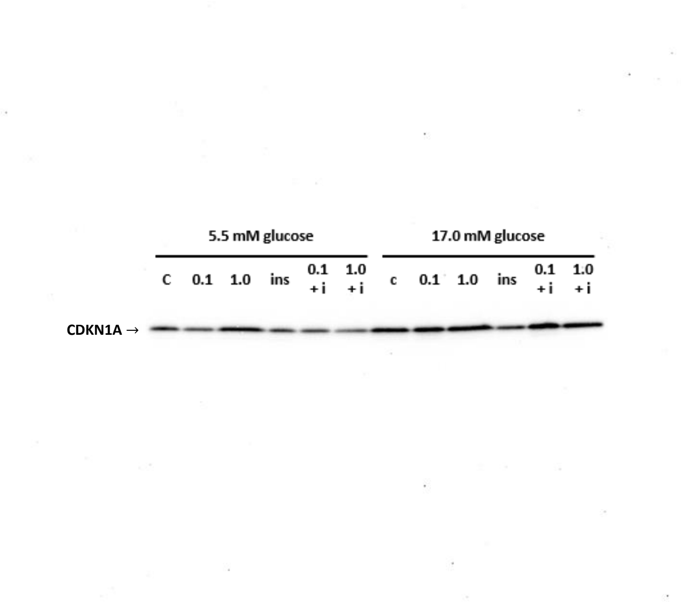

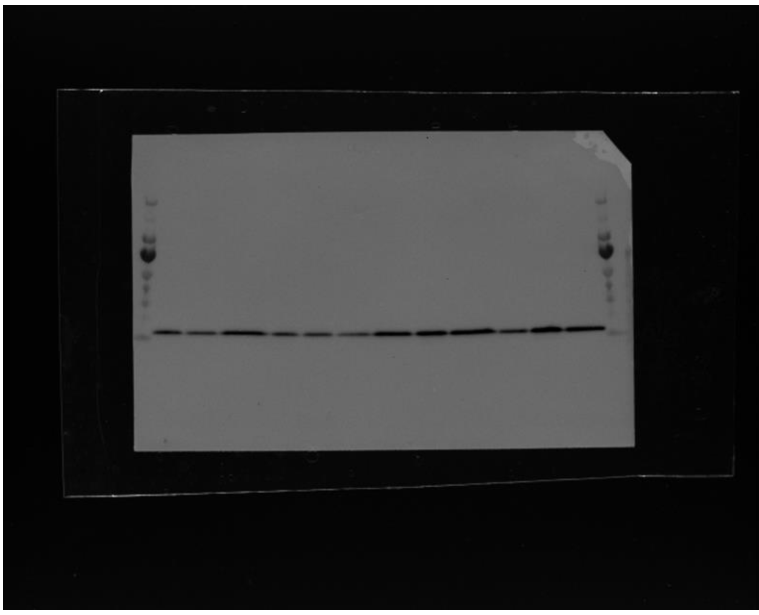

HEC-1A **COL1A1:**

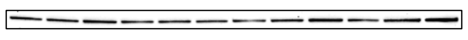

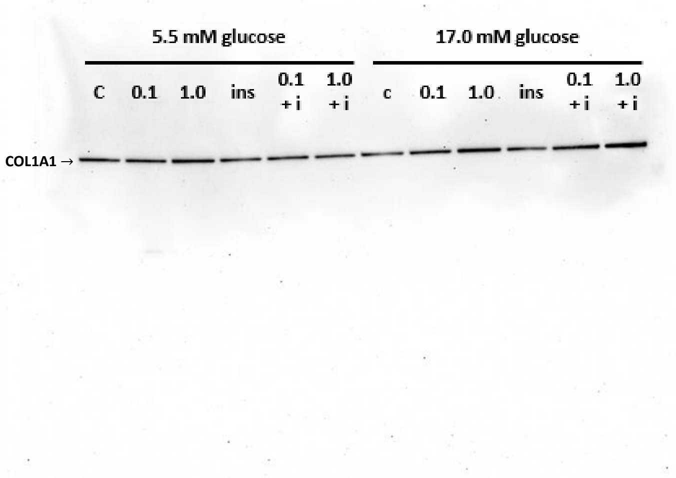

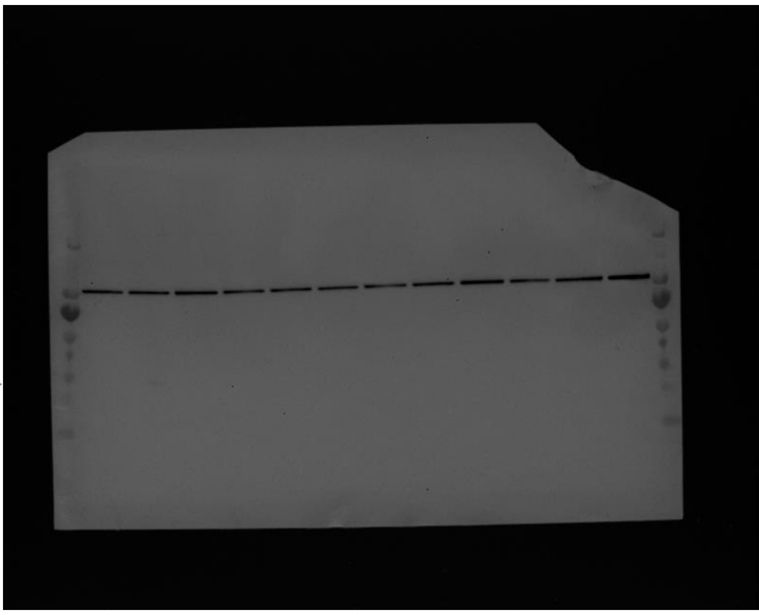

HEC-1A **PTEN:**

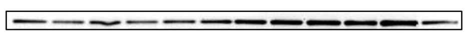

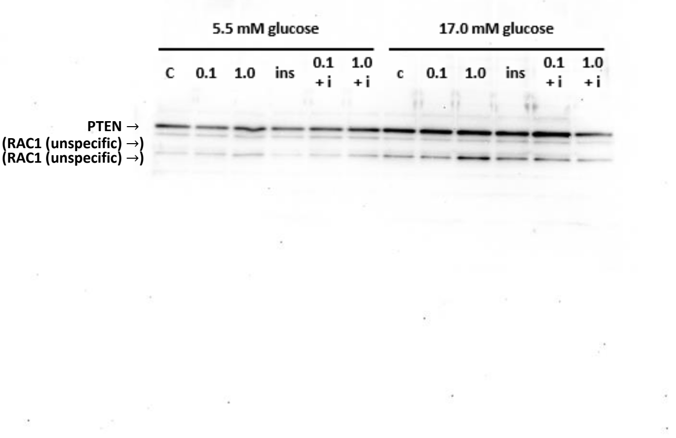

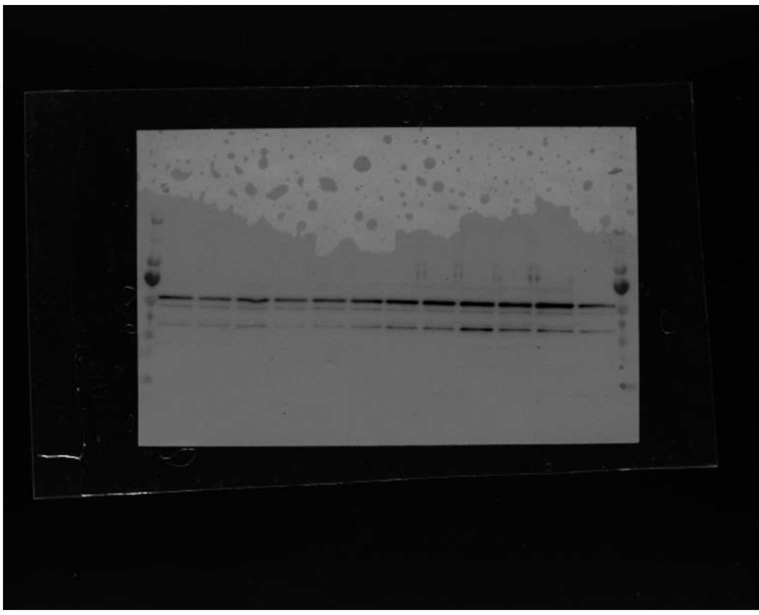

HEC-1A **MMP9:**

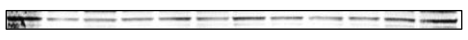

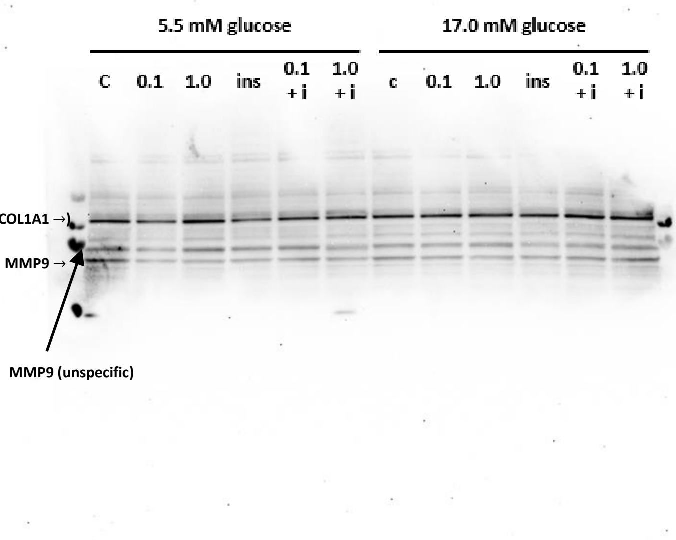

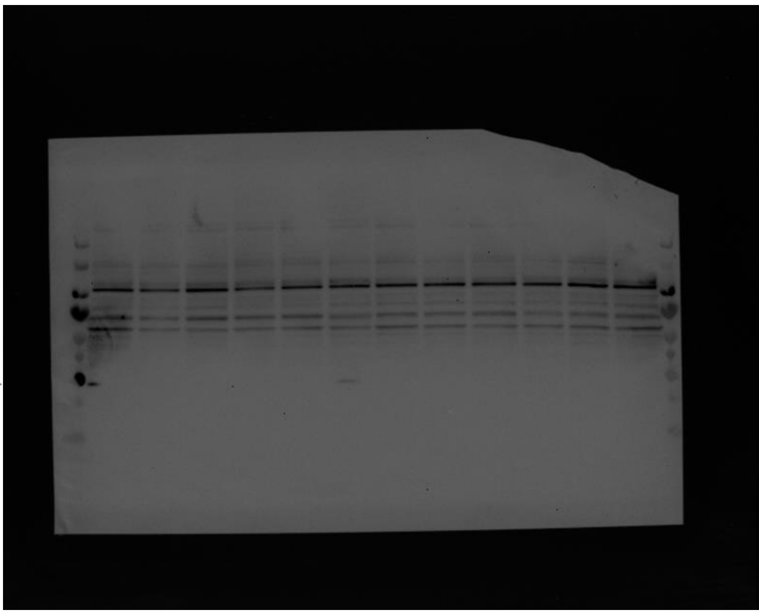

HEC-1A **TIMP2:**

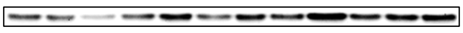

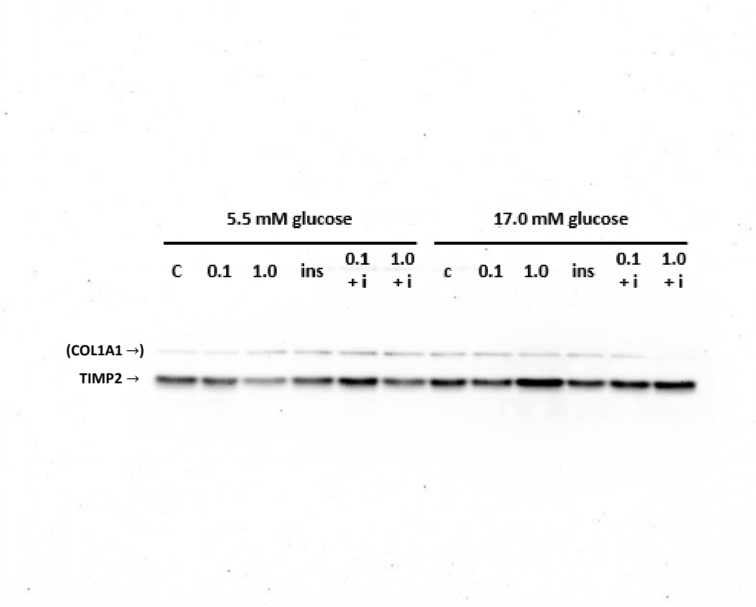

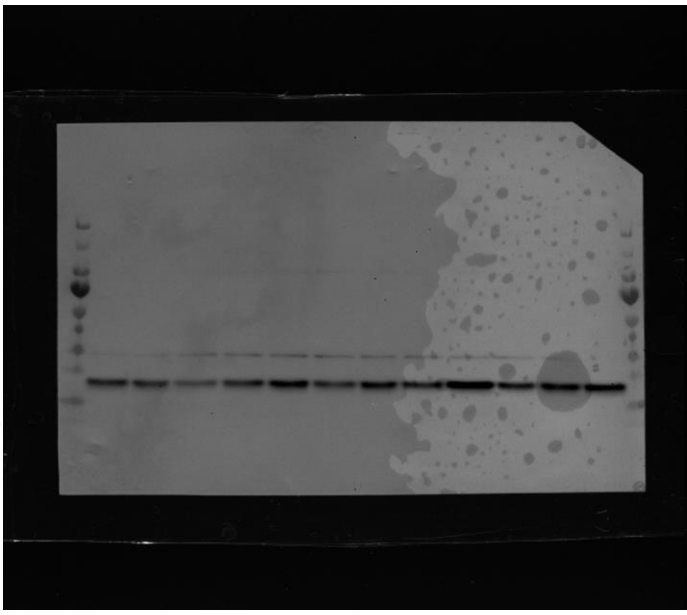

HEC-1A **TP:**

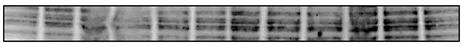

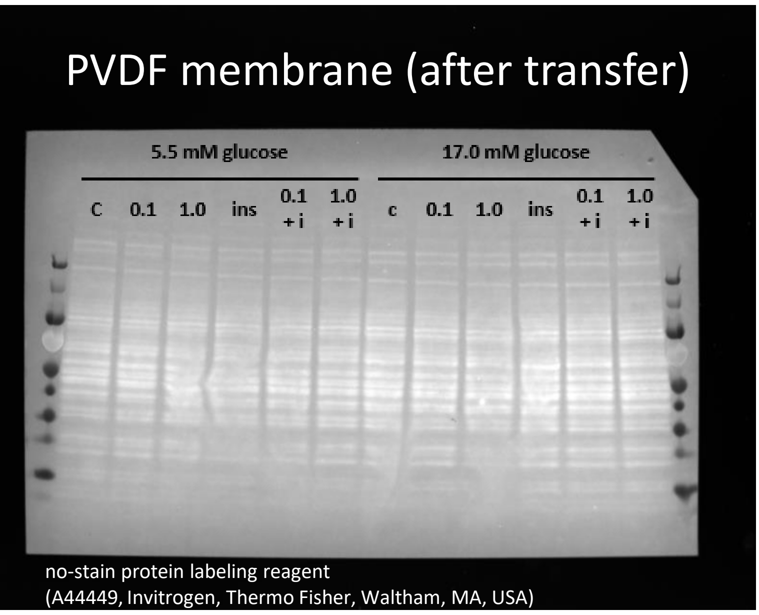

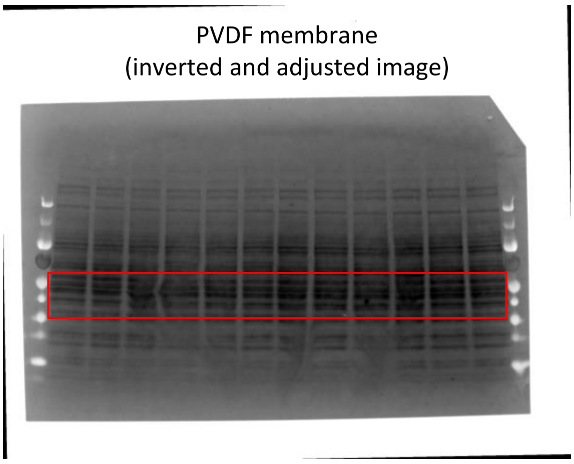

Section shown in Figure 3

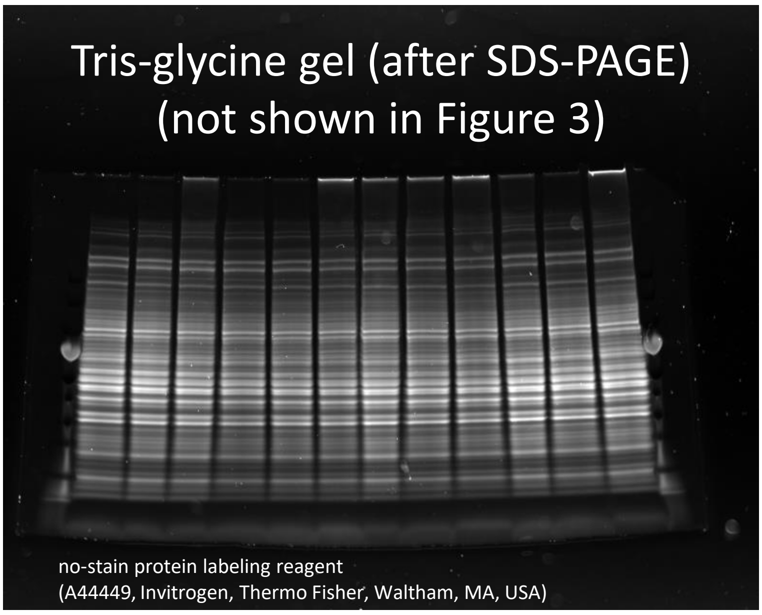

Uncropped and unadjusted representative images used for Figure 3

Chemiluminescence

Marker Overlay

Ishikawa

BCL2L11:

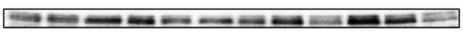

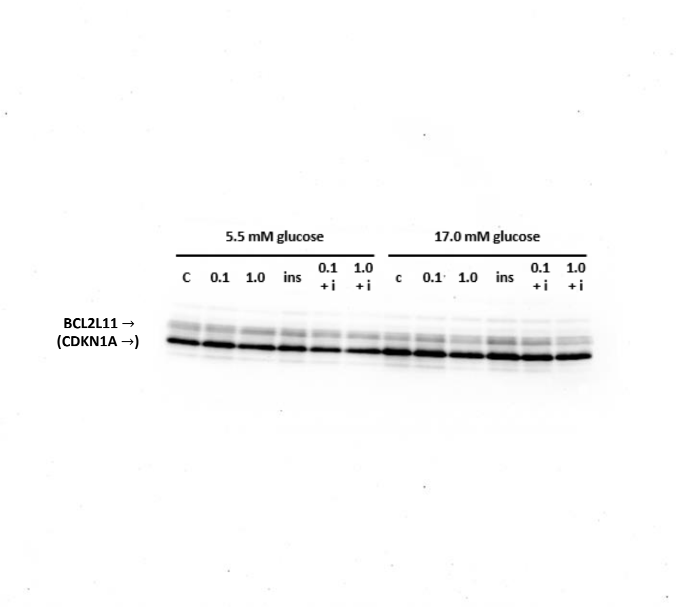

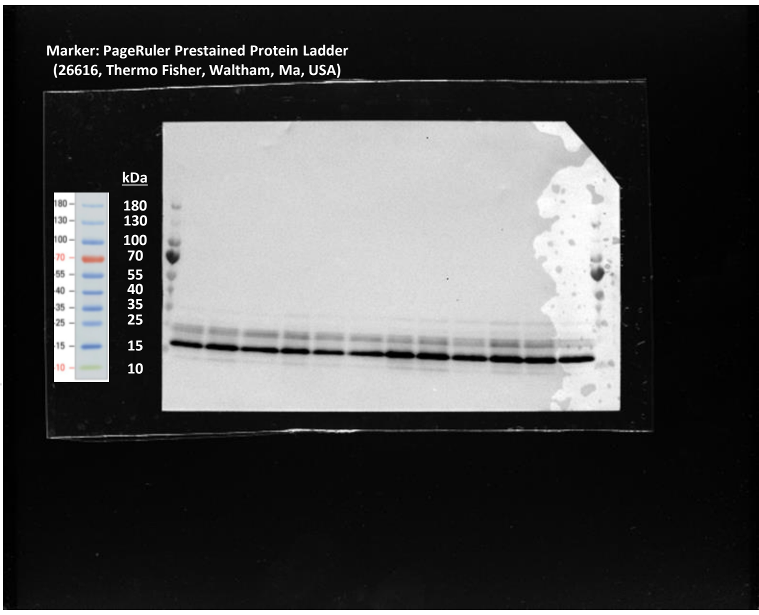

Ishikawa

CDH1:

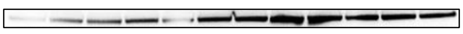

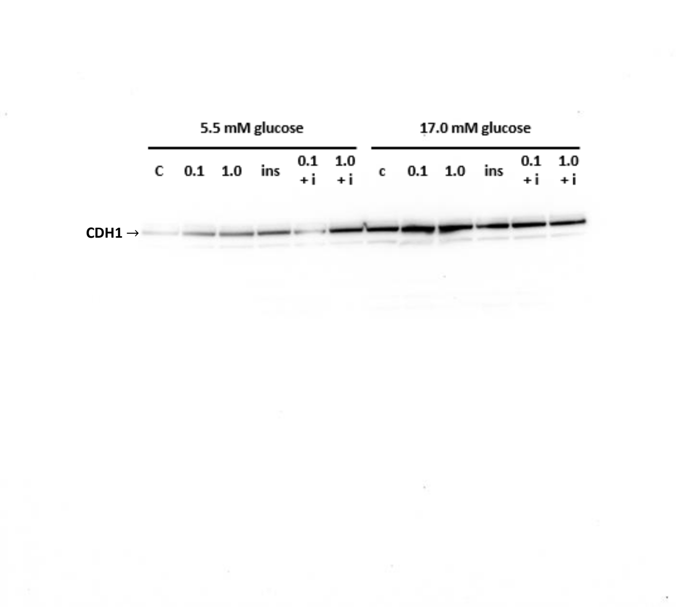

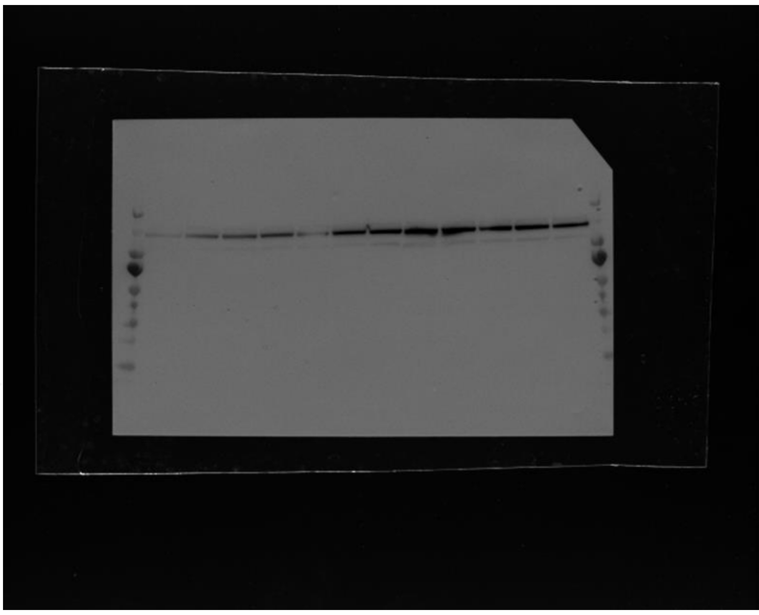

Ishikawa

CDKN1A:

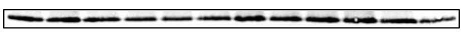

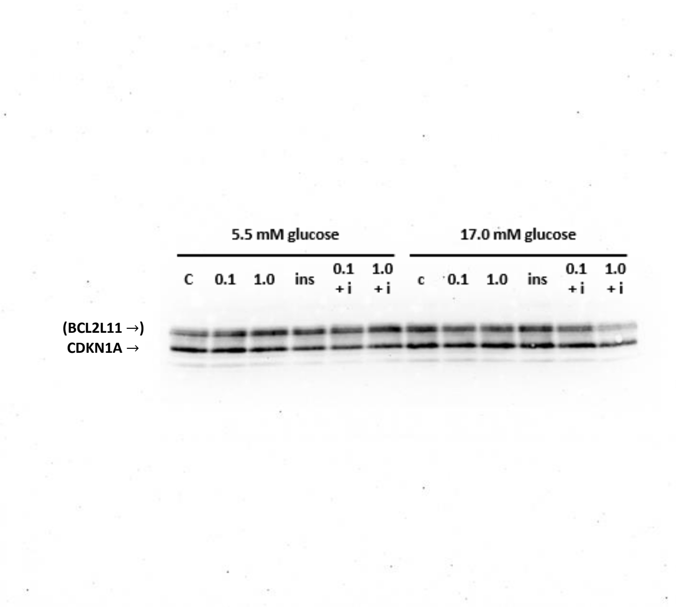

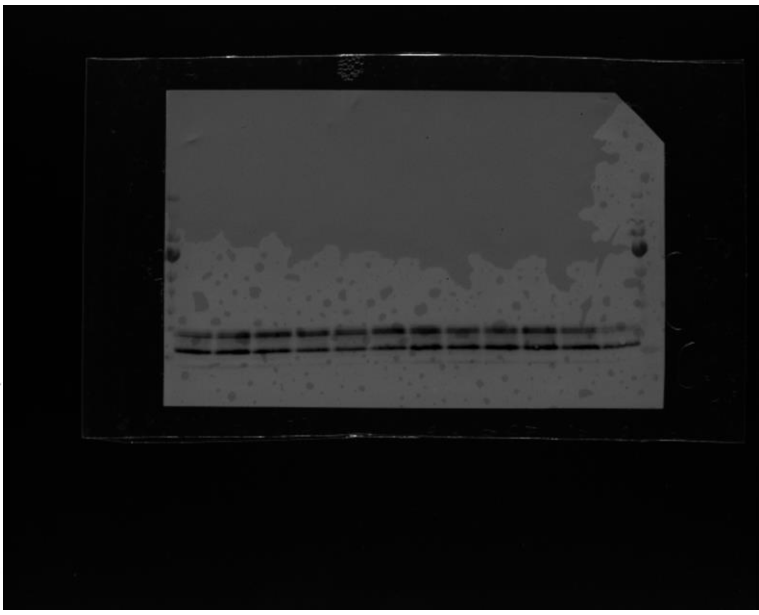

Ishikawa **COL1A1:**

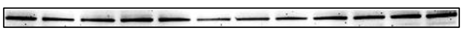

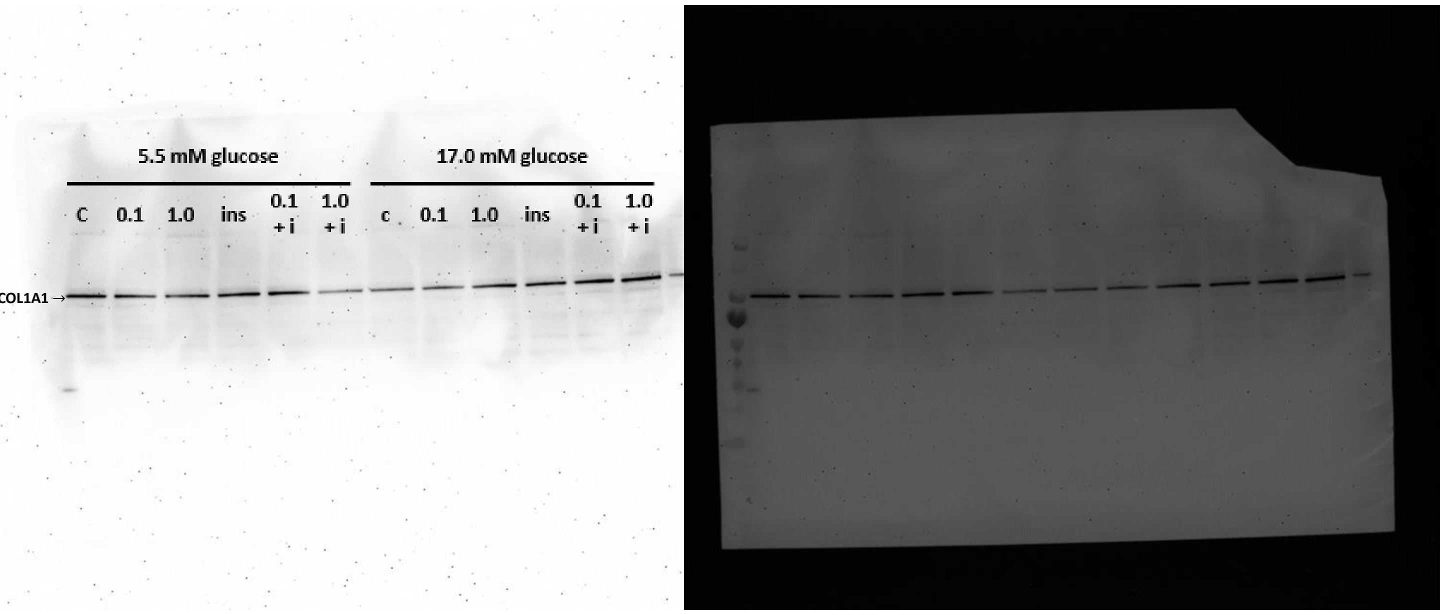

Ishikawa **PTEN:**

not detectable

HEC-1A **MMP9:**

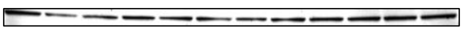

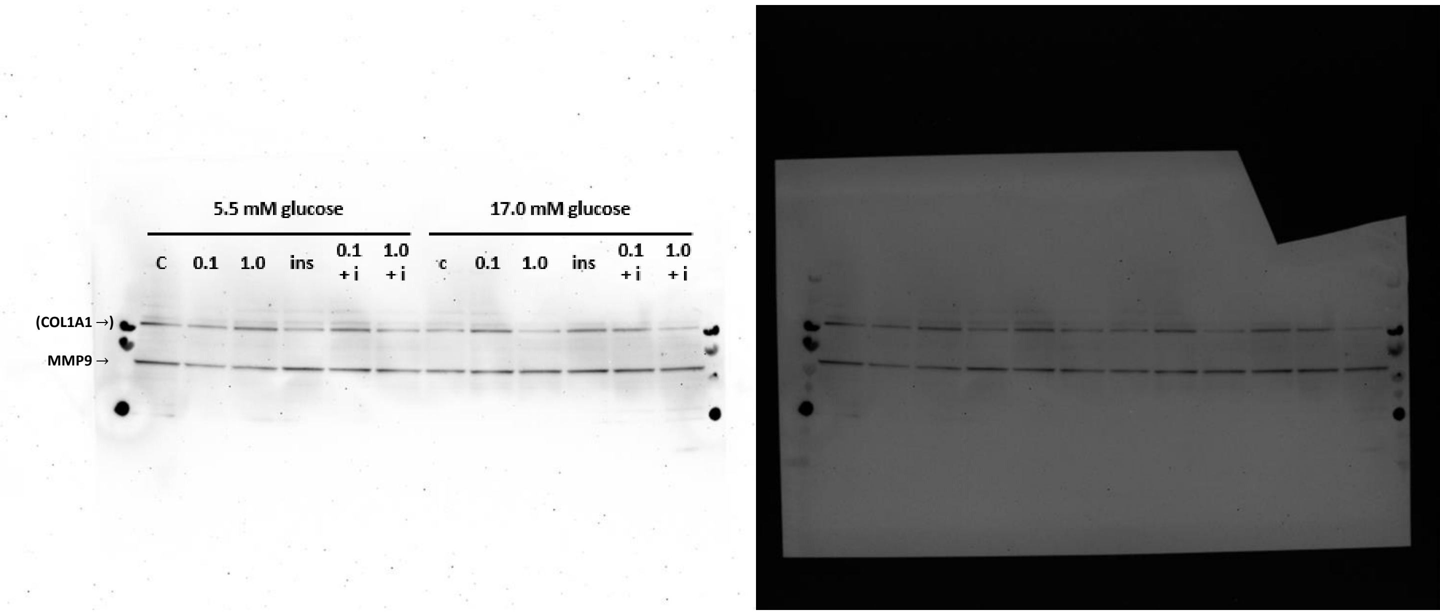

Ishikawa

TIMP2:

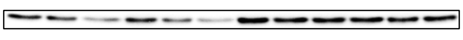

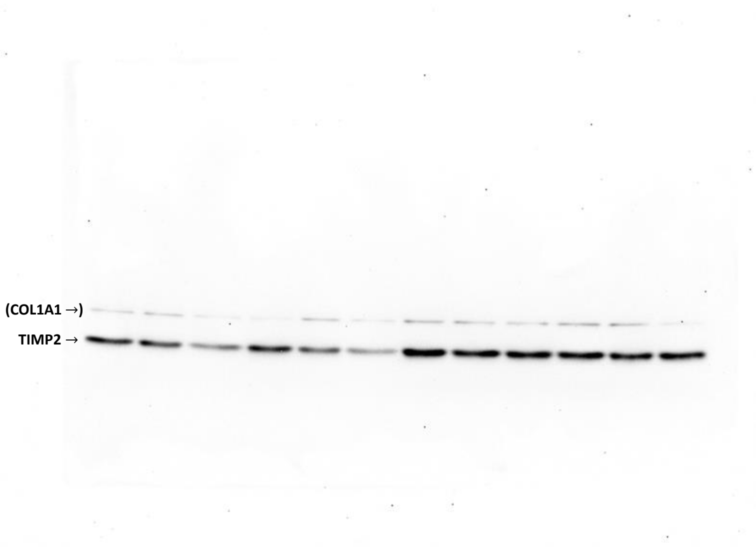

marker overlay:
not documented

Ishikawa

TP:

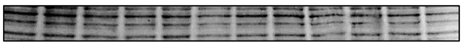

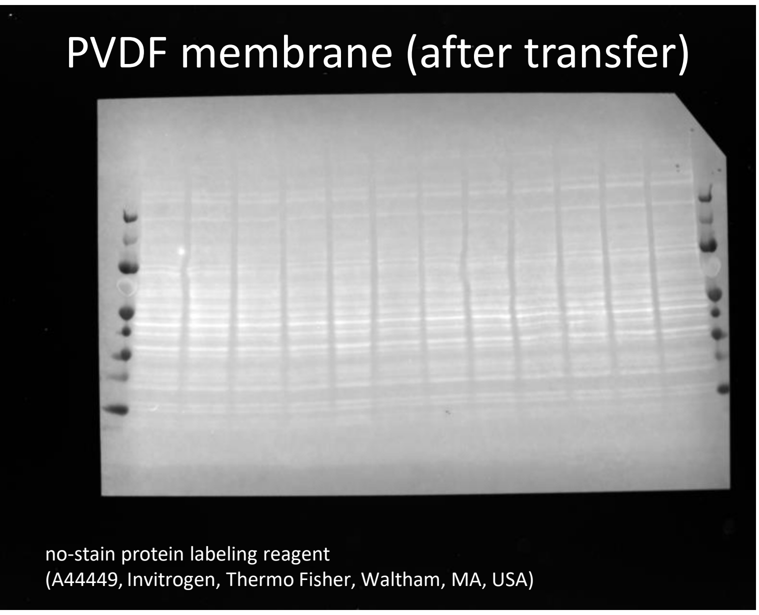

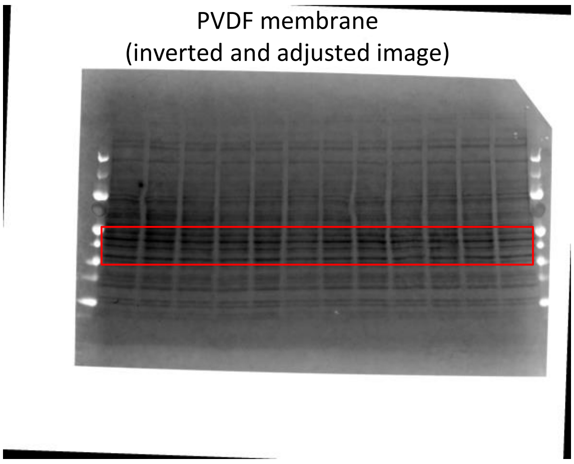

Section shown in Figure 3

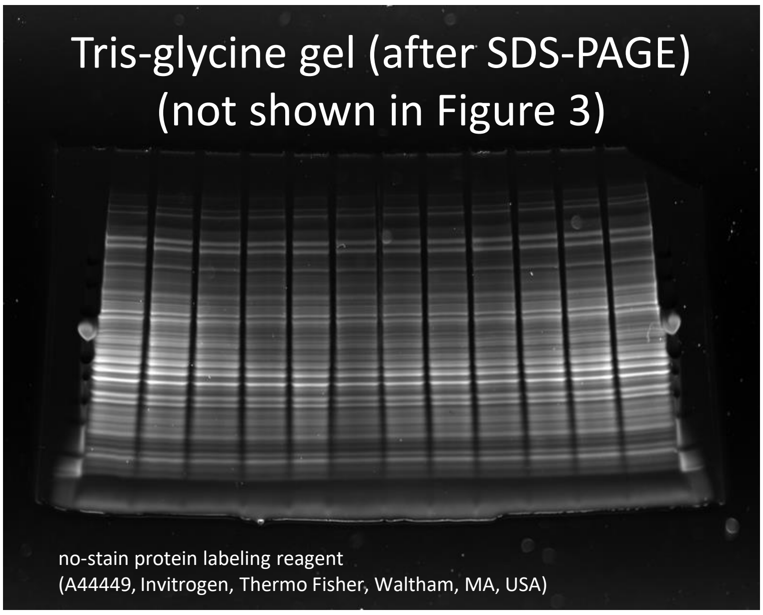

Supplement: Multimedia component 3 [file mmc3.pdf]
